# Targeting hepatic kisspeptin receptor ameliorates nonalcoholic fatty liver disease in a mouse model

**DOI:** 10.1172/JCI145889

**Published:** 2022-05-16

**Authors:** Stephania Guzman, Magdalena Dragan, Hyokjoon Kwon, Vanessa de Oliveira, Shivani Rao, Vrushank Bhatt, Katarzyna M. Kalemba, Ankit Shah, Vinod K. Rustgi, He Wang, Paul R. Bech, Ali Abbara, Chioma Izzi-Engbeaya, Pinelopi Manousou, Jessie Y. Guo, Grace L. Guo, Sally Radovick, Waljit S. Dhillo, Fredric E. Wondisford, Andy V. Babwah, Moshmi Bhattacharya

**Affiliations:** 1 Department of Medicine, Robert Wood Johnson Medical School, and; 2 Rutgers Center for Lipid Research, New Jersey Institute for Food, Nutrition, and Health, Rutgers University, New Brunswick, New Jersey, USA.; 3 Rutgers Cancer Institute of New Jersey, New Brunswick, New Jersey, USA.; 4 Department of Pathology and Laboratory Medicine, Robert Wood Johnson Medical School, Rutgers University, New Brunswick, New Jersey, USA.; 5 Section of Endocrinology and Investigative Medicine and; 6 Department of Metabolism, Digestion and Reproduction, Imperial College London, London, United Kingdom.; 7 Department of Pharmacology and Toxicology, School of Pharmacy, and; 8 Department of Pediatrics, Robert Wood Johnson Medical School, Rutgers University, New Brunswick, New Jersey, USA.; 9 Child Health Institute of New Jersey, New Brunswick, New Jersey, USA.

**Keywords:** Hepatology, Metabolism, G protein&ndash;coupled receptors, Mouse models, Obesity

## Abstract

Nonalcoholic fatty liver disease (NAFLD), the most common liver disease, has become a silent worldwide pandemic. The incidence of NAFLD correlates with the rise in obesity, type 2 diabetes, and metabolic syndrome. A hallmark featureof NAFLD is excessive hepatic fat accumulation or steatosis, due to dysregulated hepatic fat metabolism, which can progress to nonalcoholic steatohepatitis (NASH), fibrosis, and cirrhosis. Currently, there are no approved pharmacotherapies to treat this disease. Here, we have found that activation of the kisspeptin 1 receptor (KISS1R) signaling pathway has therapeutic effects in NAFLD. Using high-fat diet–fed mice, we demonstrated that a deletion of hepatic *Kiss1r* exacerbated hepatic steatosis. In contrast, enhanced stimulation of KISS1R protected against steatosis in wild-type C57BL/6J mice and decreased fibrosis using a diet-induced mouse model of NASH. Mechanistically, we found that hepatic KISS1R signaling activates the master energy regulator, AMPK, to thereby decrease lipogenesis and progression to NASH. In patients with NAFLD and in high-fat diet–fed mice, hepatic KISS1/KISS1R expression and plasma kisspeptin levels were elevated, suggesting a compensatory mechanism to reduce triglyceride synthesis. These findings establish KISS1R as a therapeutic target to treat NASH.

## Graphical abstract

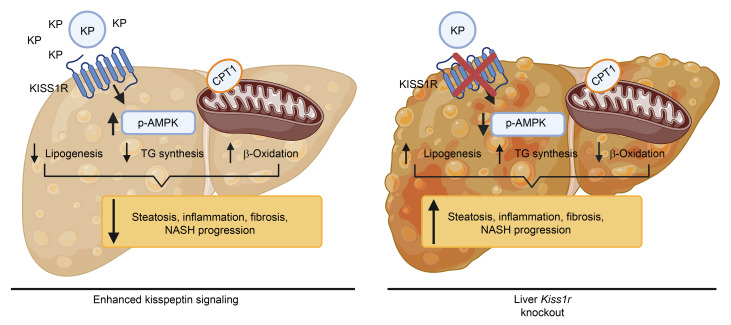

## Introduction

The liver is the principal organ involved in lipid metabolism. Dyslipidemia leads to metabolic disorders such as nonalcoholic fatty liver disease (NAFLD), which has become an increasing global public health concern, affecting approximately 25% of the population (1). In the US, NAFLD is a national epidemic that affects about 85 million adults and 8 million children, with associated annual medical costs of $103 billion (2, 3). The prevalence of NAFLD mirrors the rise in obesity and type 2 diabetes (T2D). In both adult and pediatric disease, NAFLD is more common in males than females (4). NAFLD is characterized by accumulation of liver fat (steatosis or nonalcoholic fatty liver [NAFL]), which leads to the generation of cytotoxic lipid oxidation byproducts along with other hepatic insults, which causes a progression to a chronic inflammatory state with hepatocyte injury, defined as nonalcoholic steatohepatitis (NASH). As the disease advances, a subset of patients will develop fibrosis, cirrhosis, and liver failure or hepatocellular carcinoma (HCC; ref. 5). NASH has replaced hepatitis C as the most common indication for liver transplantation (6). Currently, there are no approved pharmaceutical medicines for the treatment of NAFL or NASH.

Kisspeptins (KPs), the peptide products of the *KISS1* gene, are endogenous ligands for the KP 1 receptor (KISS1R), a Gα_q/11_ protein–coupled receptor. The KP/KISS1R signaling system is expressed both centrally in the brain and in peripheral organs, where it plays a major role in reproduction and metabolism (7, 8). In fact, liver *Kiss1* expression was found to be increased in genetic models of obesity (*db/db* and *ob/ob* mice; ref. 9). Although *KISS1* and *KISS1R* are expressed in the liver (10), a role for hepatic KISS1R signaling in lipogenesis is not known. In this study, using a high-fat diet–induced (HFD-induced) mouse model of NAFLD, we demonstrate that hepatic knockout of *Kiss1r* in mice exacerbated liver steatosis. In contrast, activation of hepatic KISS1R using a potent KP agonist (KPA) protected against the development of hepatic steatosis and was found to reduce progression to NASH and hepatic fibrosis. Mechanistically, it was observed that hepatic KP signaling activates AMPK to thereby exert its protective effects. HFD induced the expression of hepatic *Kiss1* and *Kiss1r* and increased plasma KP levels in mouse models; these observations were recapitulated using clinical samples. This study provides direct evidence that both pharmacologic and genetic interventions directed at KISS1R-mediated signaling pathway can protect against the development of NAFLD.

## Results

### Hepatic KISS1R deficiency aggravates hepatic steatosis in insulin-resistant obese mice.

**Figure 1:**
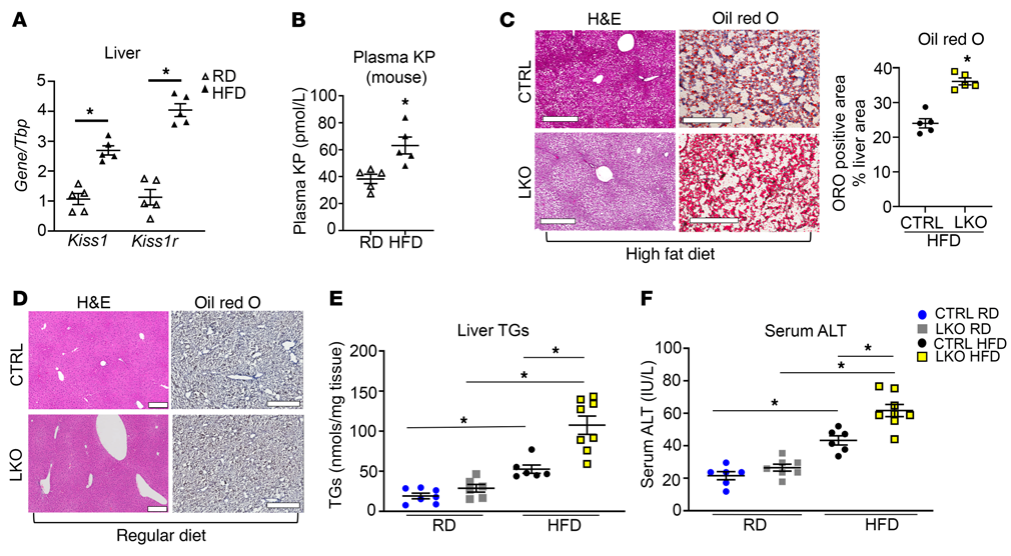
Hepatic *Kiss1r-*knockout mice exhibit increased hepatic steatosis in a diet-induced mouse model of NAFLD. (**A**) Expression of *Kiss1* and *Kiss1r* by RT-qPCR and (**B**) plasma KP levels in C57BL/6J male mice on regular diet (RD) or high-fat diet (HFD) for 12 weeks. (**C** and **D**) Representative histology of H&E- (showing steatosis) or Oil Red O–stained (showing lipids, red) liver sections. Quantification of staining is shown. Scale bars: 500 μm. No Oil Red O staining was observed in **D**. (**E**) Liver triglycerides (TGs) and (**F**) serum alanine aminotransferase (ALT) levels in control (CTRL) and hepatic *Kiss1r*-knockout (LKO) mice after 20 weeks on RD or HFD diet. Student’s unpaired *t* test or 1-way ANOVA followed by Dunnett’s post hoc test; *P < 0.05 versus respective controls.

To test whether hepatic KISS1 and KISS1R are involved in the pathogenesis of NAFLD, hepatic *Kiss1* and *Kiss1r* expression was measured in a HFD-induced mouse model of NAFLD. It was observed that after wild-type male C57BL/6J mice were fed a HFD for 12 weeks, hepatic *Kiss1* and *Kiss1r* mRNA expression significantly increased compared with that of mice maintained on regular diet (RD) (Figure 1A). In contrast, no change was observed in *Kiss1r* and *Kiss1* mRNA expression in other tissues upon administration of HFD (Supplemental Figure 1, A and B; supplemental material available online with this article; https://doi.org/10.1172/JCI145889DS1). KPs are secreted peptides, and the liver is a major source of KPs (9). Plasma KP levels were measured in C57BL/6J mice fed either RD or HFD. HFD-fed mice had significantly increased circulating KP levels compared with mice on RD (Figure 1B). Next, to investigate a role for KISS1R in regulating hepatic lipid metabolism, a mouse liver-specific knockout of *Kiss1r* (LKO) was generated. Analysis of the LKO mice showed that *Kiss1r* expression, but not *Kiss1,* was significantly reduced in the liver (Supplemental Figure 1, C and D). *Kiss1r* expression was unaffected in other metabolic organs under HFD conditions (Supplemental Figure 1C).

HFD for a duration of 20 weeks, but not RD, induced steatosis in LKO mice (Figure 1, C and D) and resulted in an increase in liver triglycerides (TGs), compared with controls (Figure 1E). Serum alanine transaminase (ALT) levels were significantly elevated in the HFD-fed LKO group compared with HFD-fed controls, suggesting enhanced HFD-induced hepatocellular injury in LKO mice (Figure 1F). Importantly, this phenotype is not due to differences in testosterone levels in HFD-fed LKO mice and controls, because these were not significantly different (Supplemental Figure 1E). As previously reported (11), there was a decrease in testosterone levels in the HFD-fed groups, although the decrease was not significant in our studies (Supplemental Figure 1E). Since hyperglucagonemia has been observed in NAFLD (12), we measured plasma glucagon levels and observed a nonsignificant increase in HFD-fed LKO mice (Supplemental Figures 1F). LKO mice also exhibited an increase in inguinal white adipose tissue compared with controls fed HFD, although no differences were observed in epididymal white adipose tissue between groups (Supplemental Figure 1, G and H, respectively). HFD-fed LKO mice displayed significantly increased body weights, compared with controls (Supplemental Figure 2A), and significantly reduced energy expenditure (Supplemental Figure 2C), despite showing no differences in food intake (Supplemental Figure 2B) or ambulatory activity (Supplemental Figure 2D). Respiratory ratio (RER) was significantly increased in the LKO HFD-fed groups during the light phase (i.e., resting phase of the nocturnal animals); this suggests that LKO mice fed HFD have a decrease in the use of endogenous lipids as fuel source and/or increased rate of DNL (Supplemental Figure 2E). Taken together, the increase in liver TGs in the LKO HFD-fed mice suggests that hepatic KISS1R plays a protective function against steatosis.

### Hepatic KISS1R deficiency upregulates the expression of genes involved in lipogenesis.

**Figure 2:**
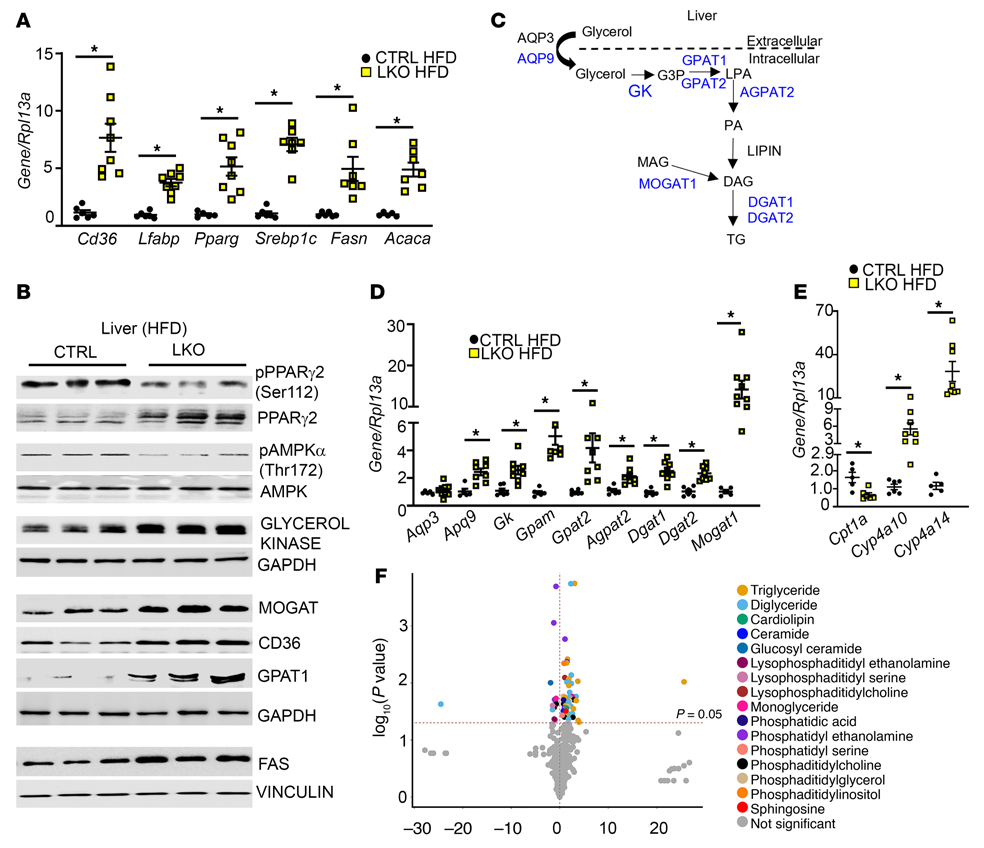
HFD-fed hepatic Kiss1r-knockout mice exhibit increased expression of genes regulating triglyceride synthesis and enhanced liver lipid levels. (**A**) Expression of indicated genes by RT-qPCR. (**B**) Representative Western blots showing expression of indicated proteins. Densitometric analyses of blots and full blots are shown in Supplemental Figure 3. (**C**) Hepatic triglyceride (TG) synthesis pathway; molecules upregulated in HFD hepatic *Kiss1r*-knockout (LKO) versus HFD CTRL livers (shown in blue). AQP, aquaporin; GK, glycerol kinase; G3P, glycerol-3-phosphate; LPA, lysophosphatidic acid; PA, phosphatidic acid; DAG, diacylglycerol; MAG, monoacylglycerol. (**D** and **E**) Expression of indicated genes by RT-qPCR. (**F**) Volcano plot showing metabolites by LC-MS in HFD livers (CTRL vs. LKO). Data are shown as the mean ± SEM. Student’s unpaired *t* test; *P < 0.05 versus respective controls.

To elucidate the mechanism underlying hepatic lipid accumulation in LKO mice, we measured the mRNA levels of hepatic regulators of fatty acid uptake (the fatty acid translocase [*Cd36*] and liver fatty acid–binding protein [*Lfabp1*]) as well as lipogenesis (*Srebp1c* and fatty acid synthase [FAS], encoded by *Fasn*) and acetyl-CoA carboxylase 1 (ACC1, encoded by *Acaca*), which catalyzes the first committed step of de novo fatty acid synthesis. It was observed that under HFD conditions, livers from LKO mice showed a significant upregulation of the expression of all genes (Figure 2A), including PPARγ, (encoded by *Pparg*), a key regulator of lipogenesis that is induced in steatotic livers of patients with NAFLD and experimental models (13, 14).

PPARγ2, in contrast to PPARγ1, is induced upon HFD feeding and is linked to the development of NAFLD (15). Protein levels of PPARγ2 (Figure 2B, top band in immunoblot) and its downstream gene targets CD36 and FAS were significantly higher in the LKO HFD-fed mouse livers compared with controls (Figure 2B and Supplemental Figure 3, A–C). PPARγ2 is negatively regulated by MAPK-dependent phosphorylation at Ser-112 (16). A decrease in PPARγ2 phosphorylation at this inhibitory site was observed in LKO livers (Figure 2B and Supplemental Figure 3D). Additionally, LKO livers exhibited suppressed phosphorylation of endogenous AMPK on the α subunit at Thr-172, a crucial phosphorylation site in the activation of AMPK (ref. 17, Figure 2B, and Supplemental Figure 3E). AMPK is a protein kinase that when activated inhibits de novo lipogenesis (DNL), by negatively regulating SREBP1 activity and its downstream gene targets, such as *Acaca* and *Fasn* (18). These data suggest that hepatic KISS1R deficiency in HFD-fed LKO mice increases lipogenesis in liver.

### Hepatic KISS1R deficiency modulates genes involved in TG synthesis and mitochondrial function.

TG synthesis (Figure 2C) requires glycerol 3-phosphate, which can be formed by glycerol kinase–dependent (GK-dependent) phosphorylation of glycerol. An analysis of the livers from the HFD-fed LKO mice revealed a significant increase in the hepatic expression of GK (a PPARγ gene target) compared with HFD control mice (Figure 2, B and D, and Supplemental Figure 3F). Previous studies have also demonstrated that HFD induces GK expression (19). Glycerol enters the liver primarily via aquaglyceroporins (AQPs), such as AQP3 and AQP9 (Figure 2C and refs. 20, 21). *Aqp9* mRNA levels were significantly upregulated in LKO HFD-fed mouse livers, whereas *Aqp3* levels remained unchanged (Figure 2D). Many enzymes regulating TG synthesis, including glycerol-3-phosphate acyltransferase (GPAT1, encoded by *Gpam*, which catalyzes the rate limiting step in TG synthesis), diacylglycerol acyltransferase 2 (*Dgat2*, acylates diacylglycerol to form TG), and monoacylglycerol acyltransferase 1 (*Mogat1*, coverts monoacylglycerol to diacylglycerol, the direct precursor of TG) (Figure 2C), were also upregulated in LKO HFD-fed mouse livers (Figure 2, B and D, and Supplemental Figure 3, G–I). MOGAT expression is also regulated by PPARγ (15). In contrast, no changes were seen in the expression of key regulators of lipogenesis or TG synthesis in the livers of LKO mice maintained on RD (Supplemental Figure 2, F–N). Taken together, this demonstrates that hepatic knockout of *Kiss1r* in HFD-fed mice results in an upregulation of genes regulating TG synthesis.

LKO HFD-fed mouse livers displayed decreased levels of mitochondrial carnitine palmitoyltransferase 1 α (CPT1α), a regulatory enzyme that transfers fatty acids from the cytosol to mitochondria prior to β-oxidation (Figure 2E). These data suggest that hepatic steatosis develops in LKO mice as a result of increased lipogenesis (Figure 2, A–D) and impaired fatty acid oxidation. In NAFLD, when cytosolic fatty acids accumulate due to impaired β-oxidation, alternative pathways in microsomes (ω-oxidation) are activated in a compensatory capacity. This was observed in LKO HFD-fed mouse livers that exhibited elevated levels of CYP4As (*Cyp4a10, Cyp4a14*) that catalyzed ω-oxidation (Figure 2E). Collectively, these data suggest that hepatic steatosis develops in LKO mice due to increased DNL and TG synthesis and impaired mitochondrial β-oxidation.

### Hepatic KISS1R deficiency alters lipidomic profiling in liver extracts.

To identify metabolic differences contributing to the distinct phenotypes observed in LKO mice under HFD conditions, a global untargeted metabolomic analysis of LKO (LKO HFD) and control (CTRL HFD) livers was conducted. This revealed that various lipids, including TG, DAG, and lysophosphatidylcholine, were significantly upregulated in livers from LKO HFD-fed mice (Figure 2F). Similar observations were also seen in patients with NAFLD and NASH (22). Livers from LKO HFD-fed mice also exhibited other changes, including high levels of ceramides, phosphatidylglycerol, and cardiolipin. The inhibition of ceramide synthesis was reported to attenuate hepatic steatosis and fibrosis, while phosphatidylglycerol, a mitochondrial phospholipid, is implicated in multiple metabolic diseases, including hepatosteatosis (22). Cardiolipin is a phospholipid that is essential for optimal mitochondrial function and alterations contribute to mitochondrial dysfunction in multiple tissues, including insulin resistance and NAFLD (23).

### Hepatic KISS1R deficiency promotes insulin resistance, hepatic inflammation, and hepatic fibrosis biomarkers.

**Figure 3:**
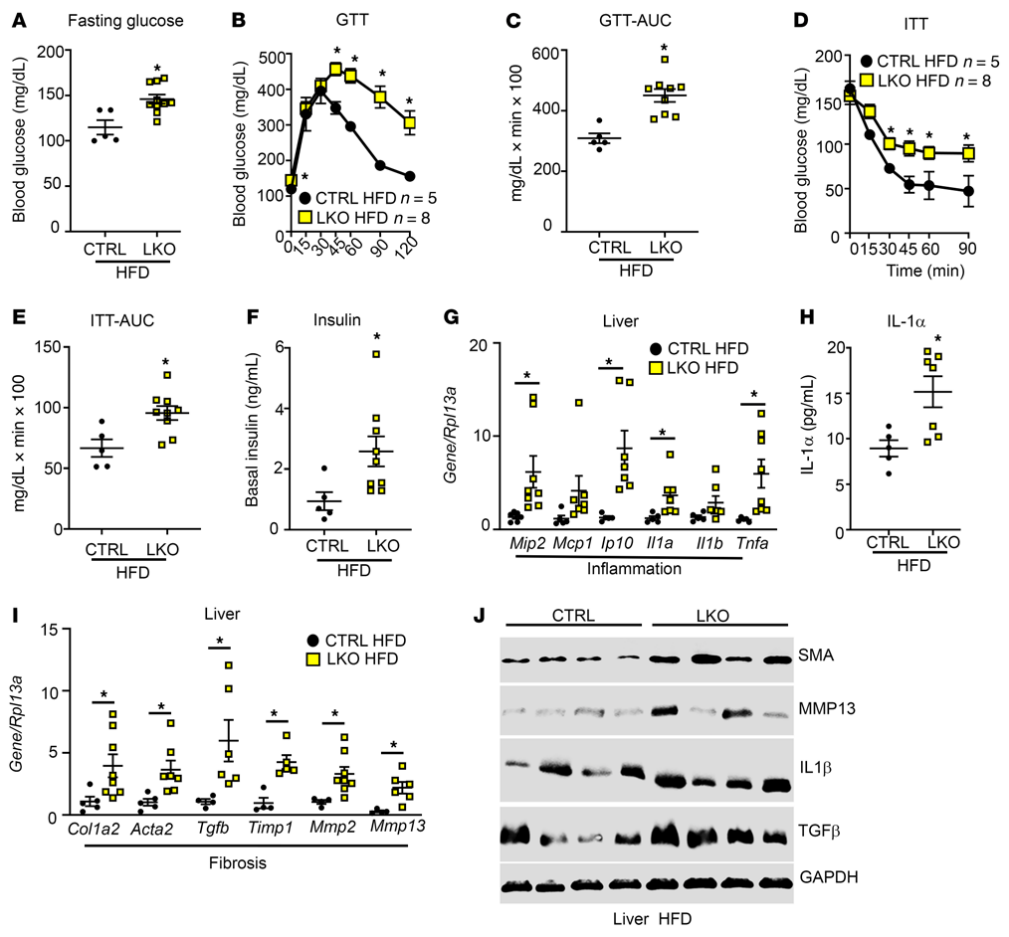
HFD-fed hepatic *Kiss1r*-knockout mice are glucose intolerant and insulin resistant, and they exhibit increased inflammation and fibrosis markers. (**A**) Fasting blood glucose levels. Blood glucose levels during (**B**) a glucose tolerance test (GTT) and (**C**) AUC of the GTT as well as (**D**) an insulin tolerance test (ITT) and (**E**) AUC of the ITT. (**F**) Basal insulin levels. (**G**) Expression of indicated genes by RT-qPCR. (**H**) Serum IL-1α levels. (**I**) Expression of indicated genes by RT-qPCR. (**J**) Expression of indicated protein by Western blot analysis. Densitometry analyses of blots shown in Supplemental Figure 3. Data are shown as the mean ± SEM. Student’s unpaired *t* test or 1-way ANOVA followed by Dunnett’s post hoc test; *P < 0.05 versus respective controls.

Because selective insulin resistance plays an important role in the pathogenesis of NAFLD, metabolic tests were performed to examine the effect of the loss of hepatic KISS1R on glucose homeostasis. Compared with HFD-fed controls, LKO HFD-fed mice had significantly higher fasting glucose levels, indicative of elevated gluconeogenesis (Figure 3A). They were also glucose intolerant (Figure 3, B and C) and insulin resistant (Figure 3, D and E). Consistent with the insulin resistance phenotype, basal insulin levels were significantly upregulated in LKO mice fed HFD, compared with controls fed the same diet (Figure 3F).

NAFL can progress to NASH, a state associated with increased inflammation, fibrosis, and oxidative stress in the liver (24). HFD feeding induces insulin resistance, liver steatosis and modest inflammation but does not cause significant hepatocyte injury or fibrosis (25, 26). We observed an upregulation of various markers involved in inflammation and early stages of fibrosis in LKO mice after 20 weeks of HFD. These included inflammatory markers associated with NASH (27–29), such as macrophage inflammatory protein 2 (*Mip2);* chemokines IFN-γ–induced protein 10 *(Ip10*) and IL-1α (*Il1a*); and proinflammatory cytokine TNF-α (*Tnfa*; Figure 3G). IL-1α serum levels were elevated in HFD-fed LKO mice compared with controls (Figure 3H). Various markers of fibrosis, such as collagen (*Col1a2*), smooth muscle actin (SMA; *Acta2*), matrix metalloproteinases (*Mmp2, Mmp13*), and *Tgfb*, were upregulated in livers from the LKO (HFD) group (Figure 3I), although at the protein level only SMA was significantly different (Figure 3J and Supplemental Figure 3, J–M). This is not surprising, given that HFD feeding alone induces modest inflammation (25, 26) and does not cause substantial hepatocyte injury or fibrosis (30). Together, these findings suggest that loss of hepatic KISS1R signaling exerts a deleterious effect on the liver by increasing hepatic steatosis and the progression to NASH.

### KISS1R agonist alleviates hepatic steatosis and metabolic deterioration in a wild-type mouse model of NAFLD.

**Figure 4:**
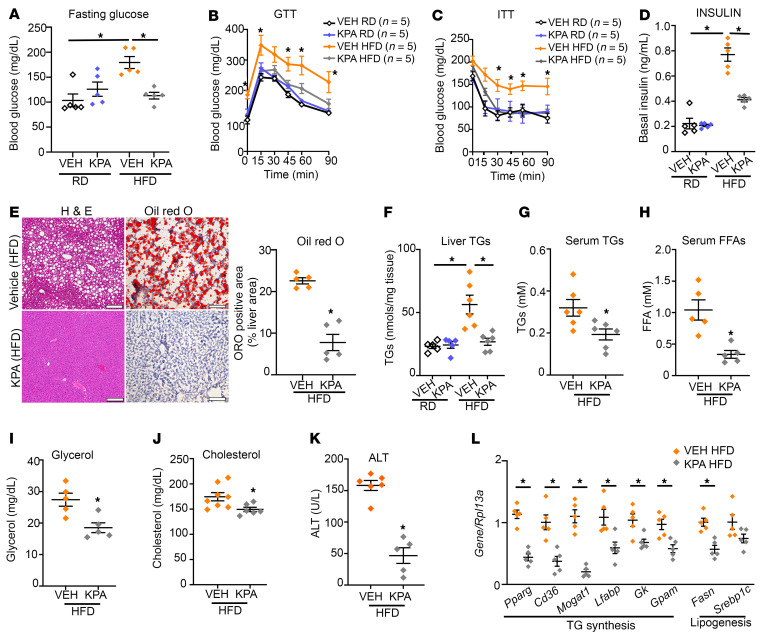
Kisspeptin analog treatment protects against insulin resistance and hepatic steatosis in HFD-fed mice. Blood glucose levels during (**A**) fasting, (**B**) GTT (2.5 weeks on treatment), and (**C**) ITT (3.5 weeks on treatment). KPA, kisspeptin agonist. (**D**) Basal insulin levels. (**E**) Representative histology of H&E- (left) and Oil Red O–stained (right) liver sections, showing lipid accumulation (red). Quantification of staining is shown. Scale bars: 200 μm. (**F**–**K**) Endpoint (11 weeks on diet) measurements of (**F**) hepatic triglycerides (TGs) and (**G**) serum TGs, (**H**) FFA, (**I**) glycerol, (**J**) cholesterol, and (**K**) ALT levels (5 weeks on treatment). (**L**) Expression of indicated genes by RT-qPCR. Data are shown as the mean ± SEM. Student’s unpaired *t* test or 1-way ANOVA followed by Dunnett’s post hoc test. *P < 0.05 versus respective controls.

Next, we determined the effect of enhanced KISS1R signaling on the development of NAFLD. Wild-type C57BL/6J mice (5–6 weeks of age) were placed on either RD or HFD for 6 weeks. Mice fed HFD gained weight (Supplemental Figure 4A) and developed insulin resistance, resulting in elevated fasting glucose levels (Supplemental Figure 4B). Mice (littermates, with similar body weights) were then infused with vehicle (PBS) or a KP analog, TAK-448 (0.3 nmol/hr, referred to below as KPA). This dose is based on published studies using KP in animal models and was adjusted based on weight (31, 32). This synthetic KP analog potently stimulates KISS1R activity in animals and humans (33–36). Mice were maintained on RD or HFD for another 5 weeks. KPA-treated HFD-fed mice had significantly lower fasting glucose levels compared with vehicle group controls (Figure 4A). Consistent with these phenotypes, HFD-fed KPA-treated mice were glucose tolerant (Figure 4B and Supplemental Figure 4C) and insulin sensitive (Figure 4C and Supplemental Figure 4D), with significantly lower basal insulin (Figure 4D) and glucagon levels (Supplemental Figure 4E), compared with HFD-fed vehicle-treated controls.

In addition to the effects of KPA treatment on improved glucose homeostasis, KPA treatment had a striking protective effect against the development of steatosis in the HFD-fed mice (Figure 4E), resulting in a significant decrease in liver TGs (Figure 4F). TGs are formed by esterification of free fatty acid (FFA) and glycerol and stored in hepatocytes. We found that serum levels of TGs, FFA, and glycerol as well as cholesterol were significantly lower in the KPA-treated group fed HFD (Figure 4, G–J). Importantly, KPA treatment significantly reduced serum ALT levels (Figure 4K), which indicated less liver injury. Among the HFD-fed mice, KPA-treated mice had a slightly lower body weight compared with vehicle controls (Supplemental Figure 4F), without change in food intake (Supplemental Figure 4G). KP signaling is a key regulator of the hypothalamic-pituitary-gonadal (HPG) axis (37). Importantly, prolonged exposure to KPA did not significantly affect the HPG axis, based on testosterone levels (Supplemental Figure 4H). KPA-treated mice showed significantly increased energy expenditure in the light phase and had lower RER in light and dark phase, suggesting that fat metabolism is enhanced in KPA-treated mice (Supplemental Figure 4, I and J) without significant changes in movement (Supplemental Figure 4K). Additionally, KPA-treated mice had significantly lower white epididymal and inguinal adipose tissue (Supplemental Figure 4, L and M).

**Figure 5:**
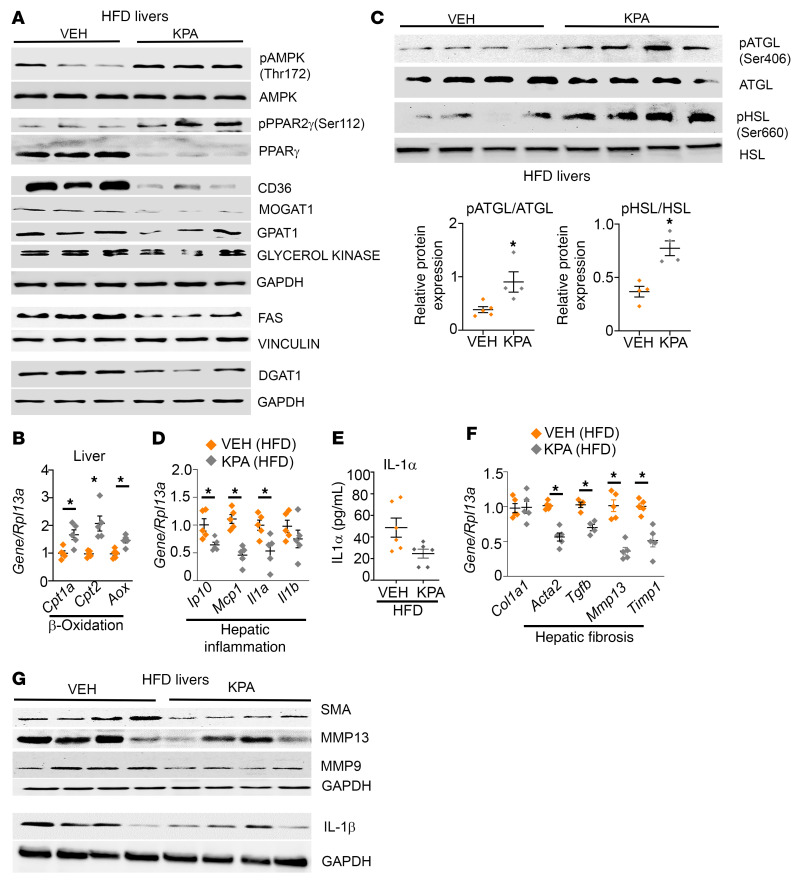
Kisspeptin analog treatment decreases markers of lipogenesis, inflammation, and fibrosis in HFD-fed mice. (**A**) Representative Western blots showing expression of indicated proteins regulating lipogenesis. KPA, kisspeptin agonist. (**B**) Gene expression by RT-qPCR. (**C**) Representative Western blots showing indicated proteins and analyses of blots. (**D**) Gene expression by RT-qPCR. (**E**) Serum IL-1α levels. (**F**) Gene expression by RT-qPCR. (**G**) Representative Western blots showing expression of indicated proteins. Densitometric analyses of blots in **A** and **G**; complete unedited blots are shown in Supplemental Figure 5. Data are shown as the mean ± SEM. Student’s unpaired *t* test; *P < 0.05 versus respective controls.

Mechanistically, KPA treatment under HFD conditions significantly reduced the hepatic expression of key regulators of TG synthesis such as PPARγ and its target genes, CD36 and MOGAT1 (Figure 4L, Figure 5A, and Supplemental Figure 5, A–C). PPARγ2 activity is negatively regulated by MAPK-dependent phosphorylation at Ser-112 (16, 38, 39). It has been established that KISS1R signaling activates MAPK (7, 40). KPA treatment stimulated phosphorylation of PPARγ2 at Ser-112, suggesting that a possible mechanism by which KISS1R regulates PPARγ2 is via MAPK (Figure 5A and Supplemental Figure 5D). Furthermore, KPA treatment induced phosphorylation of AMPK at Thr-172 (Figure 5A and Supplemental Figure 5E), which inhibits PPARγ activity and transcription (41, 42). AMPK activation also inhibits lipid synthesis by the acute inhibition of GPAT1 activity and by negatively regulating SREBP1 transcription (43). GPAT (encoded by Gpam) mRNA and protein levels were significantly reduced in KPA-treated livers (Figure 4L, Figure 5A, and Supplemental Figure 5F), which could lead to the subsequent decrease in GPAT1 activity. We also observed a decrease in DGAT1 protein expression, although it was not significant (Figure 5A and Supplemental Figure 5G). It was noted that, although the reduction in *Srebp1c* was not significant, there was a significant decrease in its downstream target, *Fasn* (Figure 4L, Figure 5A, and Supplemental Figure 5H). KPA treatment also reduced the expression of *Lfabp1* and *Gk1* (Figure 4L, Figure 5A, and Supplemental Figure 5, I and J). However, KPA treatment increased expression of *Cpt1a* and *Cpt2*, rate-limiting enzymes for mitochondrial fatty acid transportation and also increased the expression of acyl-coenzyme A oxidase (*AOX*), which regulates the rate-limiting step of peroxisomal β-oxidation of fatty acids (Figure 5B). Because hepatic lipolytic enzymes adipose TG lipase (ATGL) and hormone sensitive lipase (HSL) regulate hepatic TG metabolism by increasing lipolysis and promoting fatty acid oxidation (44), we examined whether KPA regulated the phosphorylation status of these enzymes. A significant increase in phosphorylation of both enzymes was observed in livers from KPA-treated mice, which suggests increased activity and lipolysis (Figure 5C). Thus, KPA administration in vivo appears to enhance hepatic lipolysis and mitochondrial and peroxisomal β-oxidation.

Livers from KPA-treated mice had suppressed levels of genes regulating proinflammatory markers (*Ip10, Mcp1, Il1a*) (Figure 5D). Serum levels of IL-1α were lower in KPA-treated groups (Figure 5E), although significance was not reached. IL-1β plays a major role in the progression of steatosis to steatohepatitis and liver fibrosis (45), and levels were decreased at mRNA and protein levels, although not significantly (Figure 5, D and G, and Supplemental Figure 5K). Decreases in various markers for fibrosis were observed in KPA-treated livers (Figure 5, F and G, and Supplemental Figure 5, L–N); however, HFD feeding alone did not strongly induce inflammation or establish fibrosis. In contrast, no significant differences in the expression of key regulators of lipogenesis or TG synthesis were observed in age-matched C57BL/6J mice maintained on RD, upon KPA treatment (Supplemental Figure 6). This is consistent with the lack of increase in liver TGs observed in KPA-treated mice, compared with controls maintained on RD (Figure 4F). Only GK protein levels were significantly lower in KPA-treated groups (Supplemental Figure 6, C and F), but no change in mRNA was observed (Supplemental Figure 6B). No differences were observed between control and KPA-treated groups in the regulation of glucose homeostasis under RD conditions (Figure 4, B and C). Next, to understand the specific role of hepatic *Kiss1* in NAFLD, we depleted *Kiss1* levels (Supplemental Figure 7A) by expressing AAV8-U6-mKISS1 shRNA (shKiss1) or scrambled (SCRM) controls. Surprisingly, in male mice fed HFD, depletion of hepatic *Kiss1* had no effect on steatosis, liver TGs, and the expression of key regulators of lipogenesis, TG synthesis, and inflammation (Supplemental Figure 7, A–F). Additionally, no differences in body weight or glucose homeostasis were noted (Supplemental Figure 7, G–J). Taken together, this suggests that KP critically exerts its protective effect in vivo under pathophysiological conditions by activating hepatic KISS1R to downregulate lipid synthesis via AMPK activation as well as increasing β-oxidation, thus attenuating the development of NAFLD.

### KISS1R agonist fails to protect against steatosis and NASH progression in a hepatic Kiss1r-deficient mouse model.

**Figure 6:**
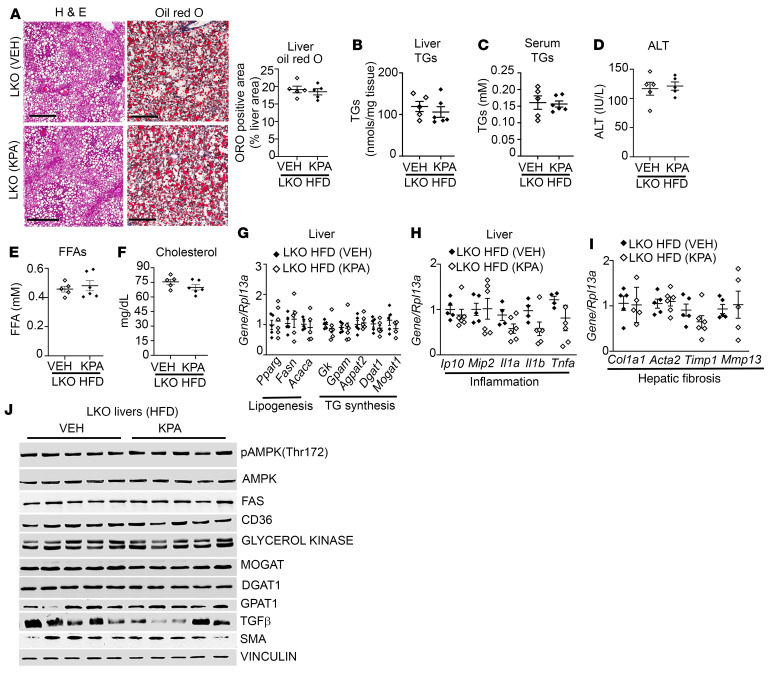
Lack of effect of kisspeptin analog treatment in hepatic *Kiss1r*-knockout mice fed HFD. (**A**) Representative histology of H&E-stained liver sections showing steatosis (left) and Oil Red O–stained (right) liver sections showing lipid accumulation. Quantification of staining is shown. Scale bars: 200 μm. KPA, kisspeptin agonist. (**B** and **C**) Levels of (**B**) liver and (**C**) serum triglycerides (TGs). (**D**–**F**) Serum (**D**) ALT, (**E**) free fatty acids (FFA), and (**F**) cholesterol levels in hepatic *Kiss1r*-knockout (LKO) mice fed HFD for 11 weeks. (**G**–**I**) Expression of indicated genes by RT-qPCR. (**J**) Representative Western blots showing expression of indicated proteins. Densitometric analyses of blots is shown in Supplemental Figure 8, F–N. Data are shown as the mean ± SEM. Student’s unpaired *t* test; *P < 0.05 versus respective controls.

Data show that activation of KISS1R by KPA had a beneficial effect, significantly reducing hepatic steatosis and decreasing NASH progression in mice fed HFD (Figures 4 and 5). In order to verify that liver-specific KISS1R signaling was crucial for mediation of the protective effects of KPA, we investigated the effect of KPA on LKO mice placed on HFD for 6 weeks prior to administration of vehicle or KPA for 5 weeks on HFD. The protective effect of KPA on steatosis was lost (Figure 6A), and levels of liver and serum TGs (Figure 6, B and C) as well as serum ALT, FFA, and cholesterol (Figure 6, D–F) were similar between vehicle and KPA-treated LKO mice. No differences were observed between the 2 groups for body weight, glucose homeostasis, or adiposity (Supplemental Figure 8, A–E). Furthermore, there were no significant differences in the expression of key regulators of lipogenesis, TG synthesis, inflammatory, and fibrosis markers (Figure 6, G–J, and Supplemental Figure 8, F–N). This provided “on-target” confirmation that the protective effect on steatosis and NASH progression is due to direct hepatic *Kiss1r* signaling by regulating these key metabolic pathways.

### KISS1R agonist fails to protect against steatosis and NASH progression in hepatic AMPK-depleted mice.

**Figure 7:**
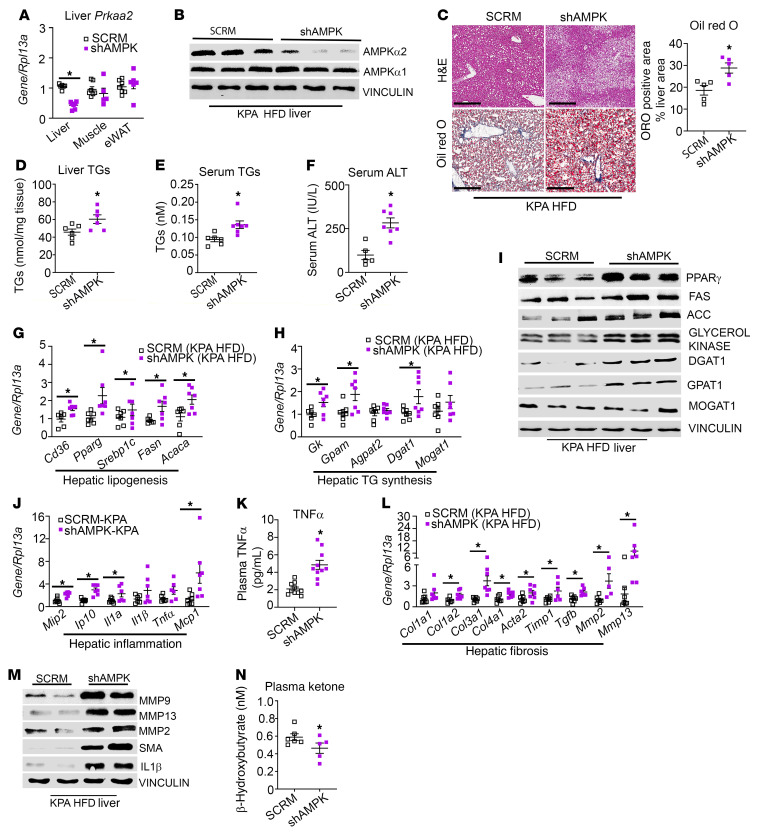
Lack of protective effect of kisspeptin agonist on steatosis and NASH progression in hepatic AMPK-knockout mice fed HFD. C57BL/6J mice fed HFD were injected with either AAV8-U6-M-PRKAA2 shRNA (shAMPK) or AAV8-U6-M-SCRM shRNA (SCRM) prior to kisspeptin agonist (KPA) treatment. (**A**) Expression of *Prkaa2* (encoding AMPKα2) by RT-qPCR. (**B**) Representative Western blot showing expression of AMPK isoforms. (**C**) Representative histology of H&E-stained (left) liver sections and Oil Red O–stained (right) liver sections. Quantification of staining is shown. Scale bars: 400 μm. (**D**) Liver and (**E**) serum triglycerides (TGs). (**F**) Serum ALT levels. (**G**, **H**, **J**, and **L**) Expression of indicated genes by RT-qPCR. (**I** and **M**) Representative Western blots showing expression of indicated proteins. (**K**) Plasma TNF-α levels and (**N**) ketone levels. Densitometric analyses of blots shown in Supplemental Figure 9; complete unedited blots are shown in Supplemental Figure 10. Data are shown as the mean ± SEM. Student’s unpaired *t* test; *P < 0.05 versus respective controls.

To dissect the in vivo contribution of hepatic AMPK in mediating the protective effects of KP signaling in NAFLD, the expression of AMPKα2 was depleted in the livers of C57BL/6J mice fed HFD (Figure 7, A and B, and Supplemental Figure 9, A and B). This isoform has been shown to critically control hepatic lipogenesis (46). Mice were placed on HFD for 4 weeks and then injected with either AAV8-U6-M-PRKAA2 shRNA (shAMPK) or SCRM controls. Mice were maintained on HFD for another 3 weeks before KPA was administered to the SCRM and shAMPK groups for 6 weeks in addition to HFD. There were no significant changes observed in body weight (Supplemental Figure 9C) or energy expenditure, RER, or movement (data not shown) between the 2 groups. However, in contrast to KPA-treated controls, there was a marked increase in steatosis and Oil Red O staining in the livers from the KPA-treated shAMPK mice (Figure 7C). Liver TGs, serum TGs, and ALT levels were significantly elevated in shAMPK group (Figure 7, D–F). Various markers for lipogenesis and TG synthesis were upregulated in the shAMPK cohort (Figure 7, G–I; Supplemental Figure 9, D–J; and Supplemental Figure 10). Several inflammatory genes, such as *Mip2*, *Ip10*, and *Il1a* (Figure 7J), as well as TNF-α serum levels (Figure 7K) and IL1-β protein levels (Figure 7M, Supplemental Figure 9K, and Supplemental Figure 10C) were increased in the KPA-treated shAMPK group. Markers for fibrosis, such as MMP2, MMP9, and MMP13, and SMA were markedly elevated in KPA-treated shAMPK mice (Figure 7, L and M, and Supplemental Figure 9, L–O). However, significant differences were not observed in *Col1a1* mRNA levels (Figure 7L), and collagen 1 protein was undetected by Western blot analysis (data not shown). This is not surprising, because 13 weeks of HFD is not sufficient to fully establish fibrosis (30). Levels of plasma ketone bodies serve as a surrogate marker for hepatic β-oxidation, and liver-specific AMPKα2 deletion decreases plasma ketone levels (46). KPA-treated shAMPK mice displayed a decrease in plasma ketone levels compared with KPA-treated controls (Figure 7N). Taken together, these data demonstrate that AMPK plays an essential role in mediating the protective effect of KPA in steatosis and in reducing the progression to NASH.

### KISS1R agonist alleviates NASH in diet-induced animal model of nonalcoholic liver disease mice.

**Figure 8:**
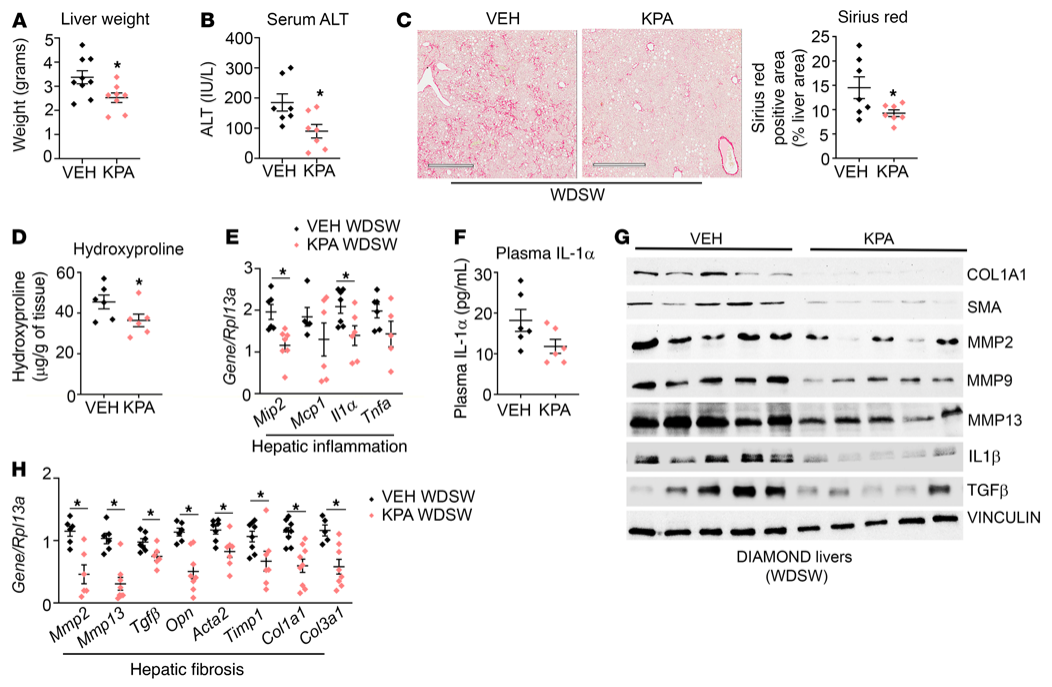
Kisspeptin agonist treatment alleviates NASH in diet-induced animal model of nonalcoholic liver disease mice. Diet-induced animal model of nonalcoholic liver disease (DIAMOND) mice maintained on a Western diet with sugar water (WDSW) for 33 weeks were treated with vehicle (PBS) or kisspeptin agonist (KPA) for 6 weeks, while they were kept on a WDSW diet. KPA, kisspeptin agonist. (**A**) Liver weight at endpoint. (**B**) Serum ALT levels. (**C**) Representative histology of Picrosirius red–stained liver section. Quantification of staining is shown. Scale bars: 500 μm. (**D**) Liver hydroxyproline levels. (**E**) Expression of indicated hepatic genes by RT-qPCR. (**F**) Plasma IL-1α levels. (**G**) Representative Western blots showing expression of indicated proteins. (**H**) Expression of indicated hepatic genes by RT-qPCR. Densitometric analyses of blots shown in Supplemental Figure 10, **A**–**G**. Data are shown as the mean ± SEM. Student’s unpaired *t* test; *P < 0.05 versus respective controls.

Diet-induced animal model of nonalcoholic liver disease (DIAMOND) mice given a high-fat Western diet and sugar water (WDSW) develop obesity, insulin resistance, dyslipidemia, and NAFL, which progresses to NASH and bridging fibrosis, closely resembling human NASH histologically (47). To determine the effect of enhanced KISS1R signaling on advanced disease, KPA or vehicle were administered for 6 weeks to DIAMOND mice that were fed WDSW for 33 weeks, while being maintained on the same WDSW diet. For this duration in DIAMOND mice, WDSW results in advanced NASH with bridging fibrosis (47). A significant decrease in liver weight and serum ALT levels was observed in KPA-treated mice (Figure 8, A and B). As expected, DIAMOND mouse livers showed signs of fibrosis based on Picrosirius red staining (Figure 8C), an indicator of collagen deposition and hepatic injury resulting in scarring (48). KPA administration significantly decreased Picrosirius red staining (Figure 8C) and also reduced the liver hydroxyproline levels (Figure 8D), indicating true collagen content (49). KPA treatment lowered the inflammatory markers (*Mip2, Il1a*), and there was a trend toward decreased serum IL-1α levels in KPA-treated mice (Figure 8, E and F). Protein levels of proinflammatory cytokine IL-1β were also significantly reduced in the KPA-treated group (Figure 8G and Supplemental Figure 11A).

**Figure 9:**
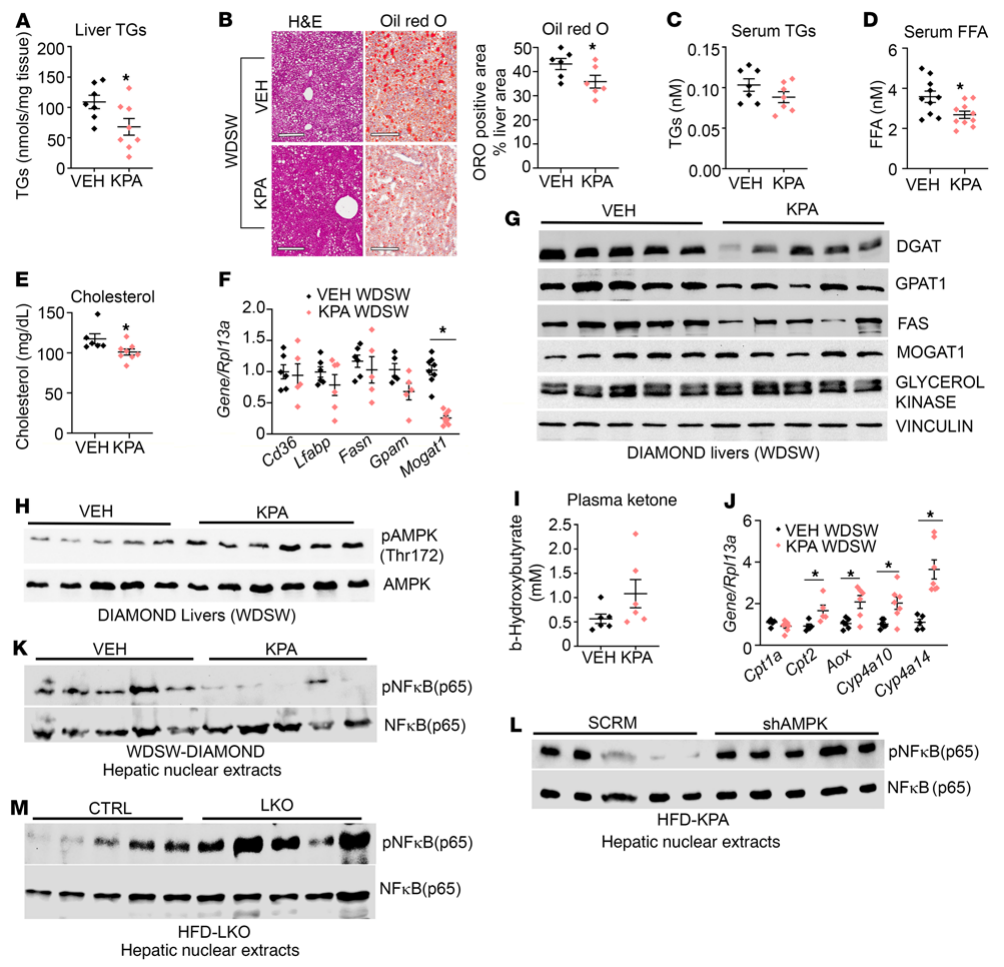
KISS1R agonist alleviates NASH in DIAMOND mice fed WDSW. Diet-induced animal model of nonalcoholic liver disease (DIAMOND) mice on Western diet with sugar water (WDSW) for 33 weeks were treated with vehicle (VEH; PBS) or kisspeptin agonist (KPA) for 6 weeks. (**A**) Liver triglycerides (TGs). (**B**) Representative liver histology for H&E and Oil Red O staining. Quantification of staining is shown. Scale bars: 500 μm. (**C**) Serum TGs levels. (**D**) Free fatty acid (FFA) levels and (**E**) cholesterol levels. (**F**) Expression of indicated genes by RT-qPCR. (**G** and **H**) Representative Western blot showing expression of indicated proteins. (**I**) β-Hydroxybutyrate levels. (**J**) Expression of indicated hepatic genes by RT-qPCR. Representative Western blot showing expression of indicated nuclear proteins in **K**. (**L**) DIAMOND mouse livers depleted of AMPK and (**M**) hepatic Kiss1r-knockout (LKO) HFD livers. Densitometric analyses of blots shown in Supplemental Figure 10, N–P. Data are shown as the mean ± SEM. Student’s unpaired *t* test; *P < 0.05 versus respective controls.

Prominent reduction in several fibrogenic genes and proteins was seen in KPA-treated DIAMOND mice, including reduction of SMA, MMPs, collagens, and TGF-β, a critical mediator of hepatic fibrosis (Figure 8, G and H, and Supplemental Figure 11, B–G). Significant decreases in liver TGs and hepatic Oil Red O staining, which marks lipids, were noted in KPA-treated DIAMOND mice (Figure 9, A and B), whereas there was a trend toward decreasing levels of serum TGs (Figure 9C). The levels of serum FFA and cholesterol were significantly reduced in the KPA-treated group (Figure 9, D and E). However, significant changes in expression of genes regulating lipogenesis were not observed, with the exception *Mogat1* (Figure 9F), although a significant reduction in MOGAT protein was not detected (Figure 9G and Supplemental Figure 11H). Protein levels of DGAT and GPAT1 were decreased in KPA-treated mice (Figure 9G and Supplemental Figure 11, I and J), although no changes between the 2 groups were observed in terms of levels of GK or FAS (Figure 9G and Supplemental Figure 11, K and L).

KPA-treated DIAMOND mice had slightly lower body weight, with no change in food intake (Supplemental Figure 12, A and B); they displayed significantly less adipose tissue (Supplemental Figure 12, C and D). These KPA-treated DIAMOND mice exhibited an increase in energy expenditure and a decrease in RER in the light phase (resting period) (Supplemental Figure 12, E and F), similar to what was observed with KPA-treated C57BL/6J wild-type mice (Supplemental Figure 4, I and J). Energy expenditure is regulated through the activity of uncoupling proteins (UCP; ref. 50), and expression of UCP1 and UCP2 in brown adipose tissue (BAT) are influenced by HFD (51). In particular, UCP2 oxidation has been shown to regulate BAT thermogenesis by favoring the utilization of FFAs (52). Interestingly, a significant increase in *Ucp2* mRNA expression was seen in BAT isolated from KPA-treated C57BL/6J mice fed HFD, whereas BAT from DIAMOND mice on WDSW displayed increases in both *Ucp1* and *Ucp2* levels (Supplemental Figure 12, G and H). PPARγ coactivator 1 (PGC1α) strongly induces UCP in BAT (53). We observed significant increases in *Pgc1a* expression in both KPA-treated models (Supplemental Figure 12, G and H). This suggests that KPA promotes brown adipocyte–mediated thermogenesis.

Similar to observations with C57BL/6J mice fed HFD (Figure 5A), KPA induced AMPK phosphorylation in DIAMOND mouse livers (Figure 9H and Supplemental Figure 11M). Increased AMPK phosphorylation and activity increase hepatic β-oxidation (54), which can be evaluated by measuring ketone levels. KPA-treated DIAMOND mice showed a trend toward increased levels of plasma ketone bodies (Figure 9I). KPA treatment significantly increased expression of *Cpt2* and *AOX*, key regulators of mitochondrial and peroxisomal β-oxidation of fatty acid, respectively, in addition to increasing the levels of *Cyp4a10* and *Cyp4a14*, which catalyze ω-oxidation of fat (Figure 9J). This is likely a potential mechanism by which hepatic lipid content decreases upon KPA administration.

It is established that AMPK signaling enhances energy metabolism, but it also represses inflammatory responses and inhibits NASH progression by suppressing liver NF-κB (55). We therefore investigated whether KPA treatment regulates hepatic NF-κB phosphorylation. A significant decrease in NF-κB phosphorylation was seen in KPA-treated livers in the DIAMOND mouse model (Figure 9K and Supplemental Figure 11N). In contrast, depletion of hepatic AMPK significantly augmented hepatic NF-κB phosphorylation in KPA-treated mice (Figure 9L and Supplemental Figure 11O). Together, these findings demonstrate an essential role for AMPK signaling downstream of KISS1R in regulating this process. Livers from HFD-fed LKO mice displayed increased hepatic NF-κB phosphorylation, compared with controls (Figure 9M and Supplemental Figure 11P). Thus, these results suggest that one mechanism by which hepatic KISS1R signaling reverses advanced NASH is by suppression of hepatic NF-κB signaling, downstream of AMPK activation. This thereby protects against HFD-induced liver steatosis and progression to NASH.

### KISS1R signaling directly activates AMPK via Gα_q/11_ and inhibits TG synthesis in isolated primary mouse hepatocytes.

**Figure 10:**
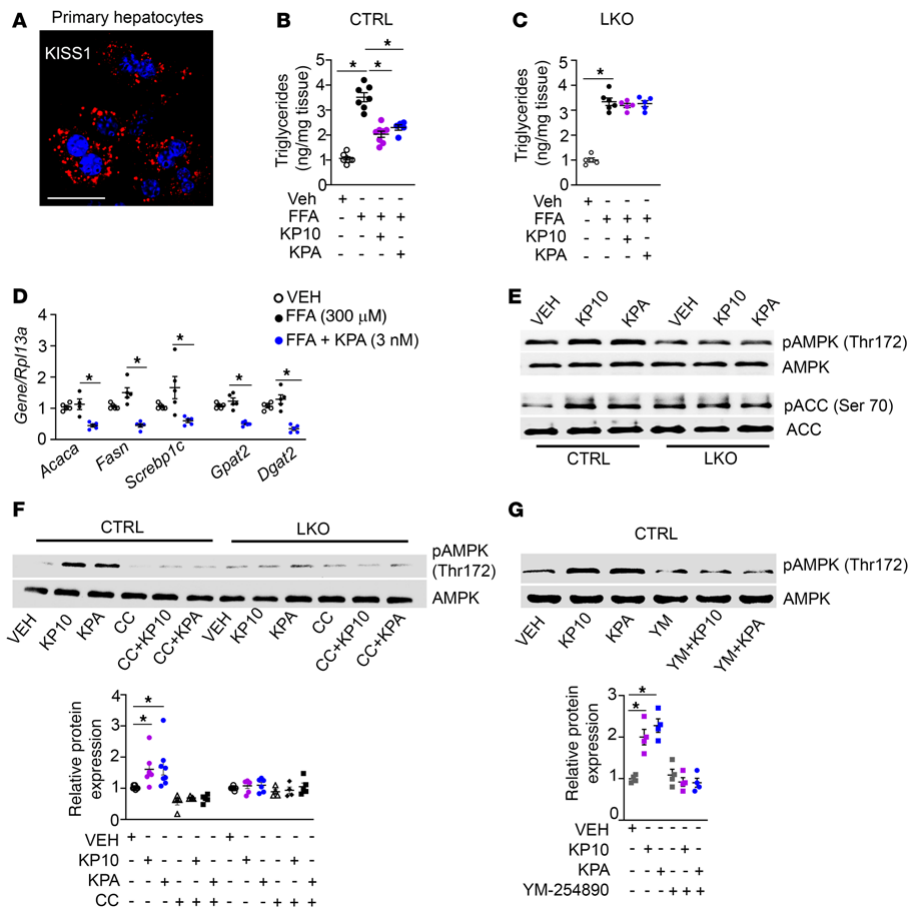
Kisspeptin inhibits triglyceride accumulation in isolated primary mouse hepatocytes. (**A**) Representative confocal image of endogenous KISS1 immunostaining in control (CTRL) hepatocytes. Scale bars: 50 μm. (**B** and **C**) Effect of KP10 (100 nM) or KPA (3 nM) on triglyceride (TG) accumulation (expressed as fold change over vehicle) in (**B**) CTRL and (**C**) hepatic Kiss1r-knockout (LKO) hepatocytes treated with oleic and palmitic acid (150 μM each). (**D**) Expression of indicated genes by RT-qPCR. (**E**) Representative Western blots of indicated proteins in hepatocytes following KP10 (100 nM) or KPA (3 nM) treatment. Quantification of blots is shown in Supplemental Figure 13, A and B. (**F** and **G**) Representative Western blots of indicated proteins in hepatocytes in the presence or absence of (**F**) compound C (CC; 10 μM) treatment, with quantification of blots, as well as in the presence or absence of (**G**) YM-25489 (YM; 3 μM), with quantification of blots. *P < 0.05 versus controls; 1-way ANOVA followed by Dunnett’s post hoc test.

Since our data revealed that KISS1R signaling inhibits steatosis in vivo, a direct effect of KP on hepatic lipogenesis was examined using isolated primary hepatocytes (56); we observed that they expressed KISS1 in a punctate pattern typical of secreted peptides (Figure 10A). Next, hepatocytes isolated from *Kiss1r*^fl/fl^ mice were cultured in the presence or absence of a mixture of FFAs (150 μM palmitate and 150 μM oleate) conjugated to BSA (57) and treated with KISS1R agonists, KP10 (100 nM) or KPA (3 nM). Treatment of FFA-loaded hepatocytes substantially decreased TG accumulation (Figure 10B). These KP concentrations were selected based on their ability to stimulate insulin secretion from isolated human pancreatic islets (58) and to activate KISS1R in vitro and in vivo (59–62). In contrast, KP failed to suppress TG levels in hepatocytes isolated from LKO mice (Figure 10C). KP treatment also reduced the expression of genes regulating DNL and TG synthesis in primary hepatocytes treated with FFAs (Figure 10D). KP stimulated phosphorylation of AMPK and its downstream target ACC in control hepatocytes (Figure 10E and Supplemental Figure 13, A and B). Phosphorylation of ACC at Ser-79 by AMPK reduces its activity, thereby inhibiting lipogenesis (63, 64). However, no change in phosphorylation of AMPK or ACC was observed in hepatocytes isolated from LKO mice (Figure 10E and Supplemental Figure 13, A and B). KP-induced AMPK phosphorylation was effectively blocked by the selective AMPK inhibitor compound C (Figure 10F) and also by the Gα_q/11_-selective inhibitor, YM254890 (refs. 65–67 and Figure 10G), in isolated CTRL hepatocytes. Taken together, this suggests for the first time to our knowledge that KP signaling via KISS1R can directly activate AMPK in isolated primary hepatocytes.

### KISS1R signaling increases fatty acid oxidation in isolated primary hepatocytes.

**Figure 11:**
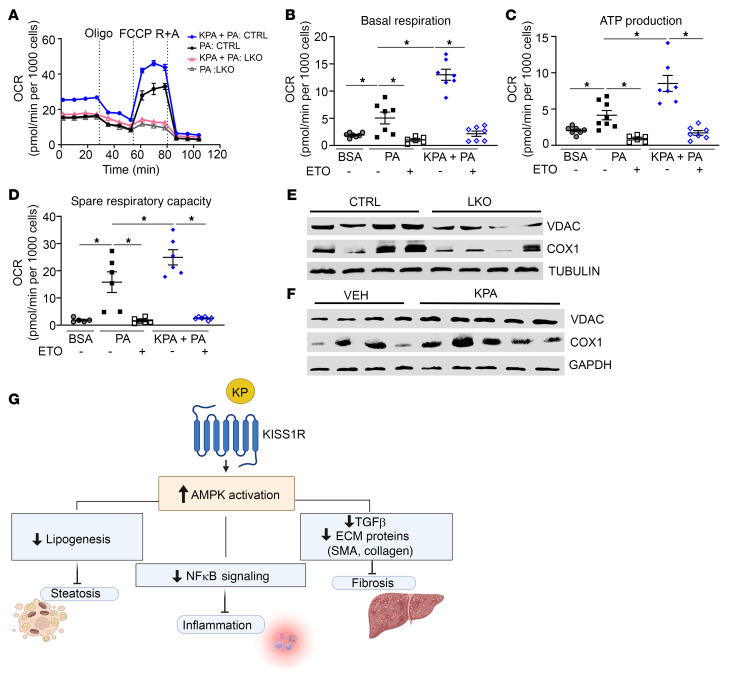
Kisspeptin increases fatty acid oxidation in isolated primary mouse hepatocytes. (**A**–**D**) Oxygen consumption rate (OCR) in hepatocytes treated with 100 μM palmitate (PA) or BSA with or without kisspeptin agonist (KPA) (3 nM) or etomoxir (ETO), an CPT1 inhibitor. Representative OCR trace using Seahorse analyzer, following sequential treatment with 2.5 μM oligomycin (Oligo); 3 μM carbonyl cyanide-4 (trifluoromethoxy) phenylhydrazone (FCCP), an uncoupler of mitochondrial oxidative phosphorylation; and 2.5 μM rotenone and antimycin A (R +A), complex I and III inhibitors. (**E**) Representative Western blot showing expression of indicated protein in primary hepatocytes isolated from control (CTRL) and hepatic Kiss1r-knockout (LKO) livers. Quantification is shown in Supplemental Figure 12, G and H. (**F**) Representative Western blot showing expression of indicated proteins in KPA-treated HFD-fed mouse livers. Quantification is shown in Supplemental Figure 12, I and J. (**G**) Schematic showing proposed signaling pathways by which KISS1R activation suppresses hepatic lipogenesis and NASH progression.*P < 0.05 versus controls; 1-way ANOVA followed by Dunnett’s post hoc test.

AMPK increases mitochondrial CPT1 activity (68). The expression of CPT1α was downregulated in LKO mice (Figure 2E), indicating impaired β-oxidation, and increased upon KPA administration in livers from HFD-fed mice (Figure 5B). Thus, we investigated whether KP regulates fatty acid oxidation. Isolated primary mouse hepatocytes were treated with palmitate (100 μM) or BSA overnight, and oxygen consumption rate (OCR) was measured using a Seahorse XFe24 Analyzer (Figure 11, A–D). KPA treatment in the presence of palmitate significantly enhanced basal OCR and ATP production compared with cells treated with palmitate alone (Figure 11, B and C, respectively). KPA treatment also increased spare respiratory capacity, which indicates a high capability to generate ATP in response to metabolic stress. In contrast, KPA failed to increase OCR in hepatocytes isolated from LKO mice (Figure 11A). Similar observations were made using human hepatic HepaRG cells, in which KPA treatment augmented OCR, in the presence of palmitate (Supplemental Figure 13, C–F). To understand mechanistically the drastic differences in OCR between control and LKO hepatocytes, mitochondrial content was examined. We found a significant decrease in mitochondrial markers voltage-dependent anion channel (VDAC), and cytochrome *c* oxidase I (COX1) in isolated hepatocytes from LKO mice (Figure 11E and Supplemental Figure 13, G and H). The expression of VDAC and COX1 was also examined in livers from C57Bl/6J mice treated with KPA; there was a significant increase in the expression of both proteins in KPA-treated liver compared with that in controls (Figure 11F and Supplemental Figure 13, I and J). This finding implicates hepatic KISS1R signaling in regulation of mitochondrial biogenesis. Taken together, findings suggest that enhanced activation of KISS1R negatively regulates hepatic lipid content by activating AMPK, which then inhibits lipogenesis and increases fatty acid oxidation. Hepatic AMPK activation downstream of KISS1R can also protect against inflammation by inhibiting NF-κΒ signaling and alleviate hepatic fibrosis by decreasing fibrogenic signaling (Figure 11G).

### KISS1/KISS1R expression and plasma KP are upregulated in humans with NAFL and/or NASH.

**Figure 12:**
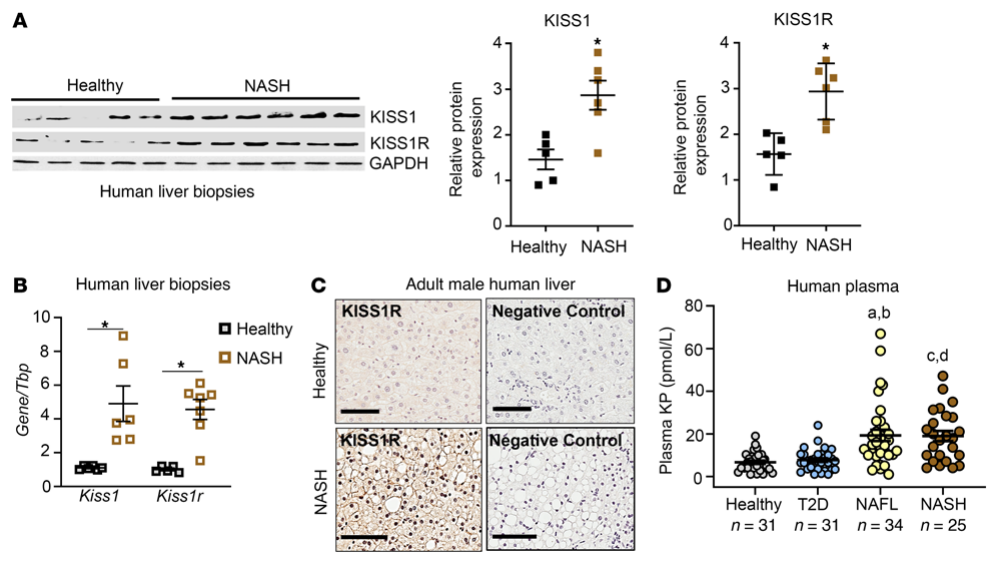
Hepatic KISS1/KISS1R expression and plasma kisspeptin levels are increased in male patients with NAFLD. (**A**) Representative Western blots and densitometric analysis of blots. (**B**) Expression of human *KISS1* and *KISS1R* by RT-qPCR. Data are shown as the mean ± SEM. Student’s unpaired *t* test; *P < 0.05 compared with controls. (**C**) Representative images showing immunostaining of endogenous KISS1R in liver. Scale bars: 80 μM. (**D**) Plasma kisspeptin (KP) levels (pmol/L; mean ± SEM) in humans. Statistical analysis done using a nonparametric Kruskal-Wallis test. Data are shown as the mean ± SEM. T2D, type 2 diabetes; NAFL, nonalcoholic fatty liver disease; NASH, nonalcoholic steatohepatitis. ^a^*P <* 0.001 for NAFL compared with healthy; ^b^*P <* 0.001 for NAFL compared with T2D; ^c^*P <* 0.001 for NASH compared with healthy; ^A^*P <* 0.001 for NASH compared with T2D.

Because we found that HFD induced the expression of hepatic *Kiss1* and *Kiss1r* and increased plasma KP levels in mouse model of NAFLD (Figure 1, A and B), we determined the clinical relevance of these findings in NAFLD. To that end, the expression of *KISS1* and *KISS1R* was examined in human liver biopsies from patients with NAFL and/or NASH. There was a significant increase in *KISS1* and *KISS1R* mRNA and protein levels in human NAFL and NASH liver samples compared with those from healthy participants (Figure 12, A and B, and Supplemental Table 1). Immunohistochemical analysis revealed enriched KISS1R expression localized to the plasma membrane and cytosol in human NAFL and NASH liver samples, compared with that in healthy liver (Figure 12C). Next, plasma KP levels were examined in male patients with NAFL and/or NASH, compared with healthy participants, as previously described (69, 70). Because the prevalence of NAFLD parallels the rise of T2D, plasma KP levels were also examined in patients with T2D. Plasma KP levels were measured in the following patient groups (see Table 1 for patient demographics): (a) healthy participants, (b) patients with T2D, (c) patients with fatty liver (NAFL), and (d) patients with NASH. The data revealed that plasma KP levels were significantly higher in patients with NAFL or NASH compared with KP levels observed in patients with T2D or healthy male participants (Figure 12D; healthy participants, 6.6± 0.8 pmol/L; patients with T2D, 7.1 ± 0.7 pmol/L; patients with NAFL,19.2 ± 2.6 pmol/L; and patients with NASH, 18.9 ± 2.4 pmol/L; mean ± SEM). This indicates that the increased plasma KP levels are associated with liver injury. Overall, the data suggest that the KISS1/KISS1R signaling pathway is enhanced in patients with liver disease, possibly as an adaptive mechanism in response to injury of the liver.

## Discussion

In this report, we provide the first evidence to our knowledge of KISS1R as a key regulator of hepatic lipogenesis. Although KISS1 and KISS1R are expressed in the liver (9, 10), their biological function in the liver was unknown. The goal of this study was to determine the role of KISS1R in the development and progression of NAFLD. We found that HFD induced the expression of hepatic *Kiss1* and *Kiss1r* and increased plasma KPs in a mouse model of NAFLD. Using LKO mice, we found that hepatic *Kiss1r* deficiency dramatically exacerbated hepatic steatosis compared with that in littermate controls fed HFD. HFD-fed LKO mice showed aggravated metabolic parameters, such as elevated levels of liver TGs, elevated fasting glucose, and insulin resistance, in addition to an increase in inflammatory and fibrosis markers. These phenotypes suggest that, under pathophysiological conditions such as obesity and insulin resistance, hepatic KISS1R plays a crucial role in suppressing the development of the NAFLD phenotype by reducing hepatic lipogenesis.

Metabolic disease, such as NAFLD, is well characterized by an alteration in glucose homeostasis, hyperinsulinemia, and hypertriglyceridemia. Under conditions of selective insulin resistance, insulin fails to suppress hepatic glucose production, while augmenting hepatic lipogenesis and TG accumulation (71). HFD-fed LKO mice display an increase in basal insulin levels, suggesting that hyperinsulinemia could contribute to the pathophysiology observed.

Despite the increase in plasma KP and hepatic *KISS1/KISS1R* levels observed in a mouse model of NAFLD or in livers of patients with NAFLD, the endogenous activation of the KISS1R signaling pathway is clearly not sufficient to safeguard against disease progression. Thus, to test the hypothesis that enhanced activation of the KISS1R signaling pathway plays a protective role in NAFLD, we used 2 HFD-fed mouse models of NAFLD, which were treated with KPA, a potent, protease-resistant KP analog (34). We found that KPA treatment in insulin-resistant wild-type C57BL/6J mice and DIAMOND mice reduced hepatic steatosis, decreased liver enzyme ALT, and reduced serum TGs, FFA, and cholesterol. Mechanistically, it was observed that KPA treatment in C57BL/6J mice decreased lipogenic regulators, although this was not consistently observed in DIAMOND mice, despite their receiving KPA treatment for same duration of (5–6 weeks). This could be due to the advanced NASH disease status of the DIAMOND mice that displayed F3 bridging fibrosis (39 weeks on WDSW), in contrast to the early disease state (i.e., DNL) observed in wild-type C57BL/6J mice (12 weeks on HFD), in addition to any differences in the background strains of mice. Hepatic fibrosis, which predicts mortality and disease severity (72), results from the activation of various pathways, such as inflammation, oxidative stress (due to mitochondrial dysfunction), and hepatic injury. As disease progresses, liver injury worsens fibrosis without changes in hepatic steatosis (73). Notably, KPA administration reduced inflammatory and fibrogenic signaling in both models and demonstrated a therapeutic effect on liver fibrosis in the DIAMOND mice.

Hepatic AMPK activity is considerably diminished in NAFL and NASH (73, 74), and this is linked to the incidence of NAFLD (75), whereas AMPK activation improves NAFL and NASH (74, 76). Our findings reveal that KISS1R activates AMPK in vivo in HFD livers and directly in isolated hepatocytes, leading to an inhibition of TG accumulation. In stark contrast, KPA failed to protect against NAFLD in livers depleted of AMPK or KISS1R. These findings demonstrate a critical protective role of KP/KISS1R signaling in the development of NAFL and its progression to NASH and fibrosis in an AMPK-dependent manner (Figure 11G).

In chronic liver disease, hepatic stellate cells are direct mediators of fibrosis (5, 24). Growth factors such as TGF-β and inflammatory cytokines, produced by other cell types such as macrophages, cause hepatic stellate cells to proliferate, transdifferentiate, become activated, and secrete excessive amounts of extracellular matrix proteins that accumulate, leading to fibrosis and cirrhosis. AMPK activation has been shown to impede hepatic fibrosis through inhibition of hepatic stellate cell proliferation by downregulating the expression of fibrogenic markers, such as SMA and TGF-β, and decreasing oxidative stress (77). In addition, hepatic AMPK activation inhibits inflammation by attenuating proinflammatory signaling pathways, such as NF-κB–mediated pathways (78). AMPK represses the nuclear localization of NF-κB to thereby inhibit the expression of NF-κB target genes. Hepatic KISS1R activation by KPA inhibited nuclear NF-κB phosphorylation in DIAMOND mouse livers, and this repression was abolished upon depletion of hepatic KISS1R or hepatic AMPK. This demonstrates a vital role for hepatic AMPK in mediating the protective effects of KPA against NAFL and its progression to NASH and fibrosis.

Fatty acids are transported into the mitochondria for β-oxidation by CPT1. AMPK increases CPT1 activity and activates FAO through phosphorylation of ACC to suppress CPT1 activity and thereby inhibit the production of malonyl-CoA, a potent allosteric inhibitor of CPT1 (63, 79). While HFD-fed LKO mice displayed decreased *Cpt1a* expression and AMPK phosphorylation, KPA treatment in HFD-fed mice increased the expression of *Cpt1a* and AMPK activation and protected against NAFLD. Using isolated primary hepatocytes, we demonstrated that KPA increased mitochondrial FAO, which was repressed upon depletion of hepatic *Kiss1r*. This could be due to KISS1R signaling influencing hepatic mitochondrial biogenesis, although this requires further investigation. Interestingly, a recent study demonstrated that KP10 administration promotes mitochondrial function in rat brain hippocampus via an AMPK-dependent pathway (80).

The link between KISS1 and mitochondrial function has been demonstrated in human melanoma cells by Welch and colleagues (81), whose pioneering work led to the initial discovery of *KISS1* as an antimetastasis gene in melanoma cells (82). This study showed that overexpression of *KISS1* in human melanoma cells resulted in increased mitochondrial biogenesis and higher oxidation of fatty acids via β-oxidation by inducing AMPK activation (81). These KISS1-mediated metabolic changes were essential for KISS1 to suppress melanoma cell invasion and metastasis (83). KISS1 functions as a metastasis suppressor gene in many cancers (84). However, KISS1R signaling in cancer appears to be context specific. Our earlier work has shown that, in triple-negative breast cancer (TNBC), KISS1R signaling promotes tumor growth and metastasis (70). When ERα is reexpressed in TNBC cells, KISS1R is downregulated, demonstrating that ERα negatively regulates KISS1R expression in TNBC (85). In native TNBC cells lacking ERα, KISS1R signaling promotes epithelial-mesenchymal transition, MAPK activation, and cancer growth and invasion (70, 86–88). The role of KISS1 in HCC has not been clearly established, although *loss* of KISS1 in human HCC is associated with an upregulation of MMP9 and increased cell invasion (89), suggesting that KISS1 may function as a metastasis suppressor in HCC.

It has been predicted that chronic infusion of KPA would result in the desensitization of the protective response; however, our observations do not provide evidence of this. We previously showed that, in cells expressing KISS1R, exposure to KP triggers rapid KISS1R desensitization and recycling (60, 90–92). However, because of the rapid nature of these events, at any given point in time, there is a KP-responsive population of receptors at the cell surface. As a result, while the receptor undergoes desensitization, the cell remains responsive to KP and exhibits prolonged signaling (60, 90–92).

The results of our human studies showed a significant increase in KISS1 and KISS1R levels in liver biopsies and elevated plasma KP levels in patients with NASH compared with those of healthy participants. In contrast, no difference in KP levels was observed in patients with T2D compared with healthy controls. These results illustrate the translational relevance of our preclinical findings, as they mirror the results that we observed using HFD-fed mice. Changes in human plasma KP levels have been reported in puberty (93) and pregnancy (94) as well as in various cancers (69, 70, 95). In fact, in proof-of-concept studies, KP has been used to identify the cause of pubertal delay in children and to treat infertility in adults (96–98). Similar to our observations, compensatory upregulation has been reported for other pathways regulating hepatic lipid homeostasis (99). These include other endocrine hormones, such as fibroblast growth factor 21 and growth differentiating factor 15, which are elevated in NAFLD and are currently being evaluated clinically. Thus, the upregulation of hepatic KISS1/KISS1R and plasma KP in NAFL and/or NASH may serve as a compensatory response that could slow down or resolve the progression of NAFLD. Our data suggest that clinical studies aimed at treating NAFL and/or NASH with KP peptides are warranted. In conclusion, this study revealed that hepatic KISS1R signaling system inhibits NAFLD via AMPK, uncovering KISS1R as a promising therapeutic target for the treatment of NAFLD.

## Methods

Further information, including that regarding methodologies and statistics, can be found in Supplemental Methods.

### Study approval.

All animal procedures were approved by the Rutgers University Institutional Animal Care and Use Committee, and its guidelines were followed in these studies. Patient blood collection was approved by the Institutional Review Board at Rutgers University and by the West London Research Ethics Committee, London, United Kingdom (12/LO/0507).

## Author contributions

All authors provided critical review of the manuscript and their approval. SG, MD, VDO, and HK performed mouse and metabolic studies, Western blotting, qPCR, and immunostaining. SG, MD, VB, and KMK performed primary hepatocyte and FAO studies, as supervised by JYG and MB. SG, S Rao, MD, and clinicians (AS, VKR, HW, PRB, AA, CIE, PM, and WSD) performed human studies. *Kiss1r*^fl/fl^ mice were generated by S Radovick, and *Kiss1r*^Alb-Cre^ mice were generated by AVB. GLG and FEW provided study design, interpretation, and resources. AVB and MB conceptualized and designed the study and provided analysis, data interpretation, and resources. MB supervised the study and wrote the manuscript with SG.

## Supplementary Material

Supplemental data

## Figures and Tables

**Table 1:**
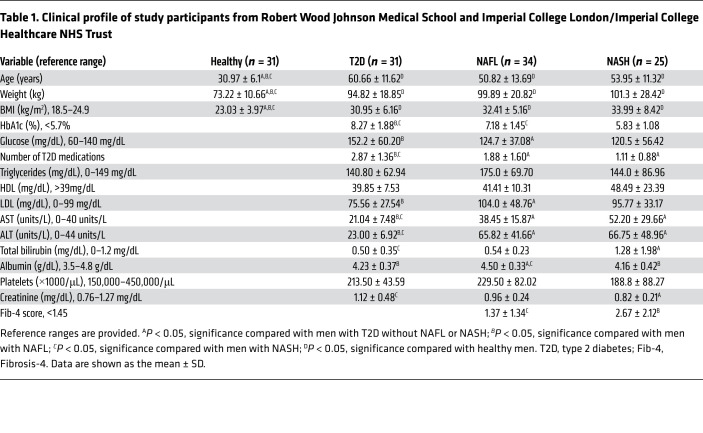
Clinical profile of study participants from Robert Wood Johnson Medical School and Imperial College London/Imperial College Healthcare NHS Trust

## Version 1 03/29/2022

In-Press Preview

## Version 2 05/16/2022

Electronic publication
