# Supplementary material for: Targeting hepatic kisspeptin receptor ameliorates nonalcoholic fatty liver disease in a mouse model
Source: J Clin Invest. 2022 May 16;132(10):e145889. doi: 10.1172/JCI145889 (PMC9106350; doi:10.1172/JCI145889)
Supplement: Supplemental data [file jci-132-145889-s131.pdf]

SUPPLEMENTAL DATA

METHODS

Animals Studies

All animal procedures were approved by Rutgers University according to guidelines established by the Institutional Animal Care and Use Committee. All experiments were conducted with male mice. *Kiss1r*$^{Alb\text{-}Cre}$ knockout mice (liver knockout: LKO) were generated by crossing a male *Alb-Cre*$^{+/-}$ *to* a female *Kiss1r*$^{fl/fl}$. *Alb-Cre* mice were purchased from Jackson Laboratories (Stock No. 003574). The *Kiss1r*$^{fl/fl}$ mouse was generated as previously described (99). The F1 generation was *Kiss1r*$^{fl/+}$, *AlbCre*$^{+/-}$ or Kiss1r$^{fl/+}$, *AlbCre*$^{-/-}$. The *Kiss1r*$^{fl/+}$, *AlbCre*$^{+/-}$ was back crossed to the *Kiss1r*$^{fl/fl}$ to generate *Kiss1r*$^{fl/fl}$, *AlbCre*$^{+/-}$ (LKO) and *Kiss1r*$^{fl/fl}$ (littermate controls). Male LKO mice and littermate controls (6 weeks old) were placed on regular diet (RD) or high fat diet (HFD) for 20 weeks. C57BL/6J mice (5-6 weeks old) were purchased from Jackson Laboratories (Stock No. 000664). These mice were used for KPA administration studies (see below) and isolation of primary hepatocytes. For knock down of hepatic *Kiss1,* C57BL/6J albino mice (8 weeks of age) were maintained on HFD for 10 weeks and then injected with AAV8-U6-Scrambled-shRNA or AAV8-U6-mKISS1-shRNA ($5x10^{11}$ genome copies/mouse) purchased from Vector Biolabs); mice were maintained on HFD diet for another 4 weeks. All mice were housed in a pathogen-free barrier facility maintained on a 12-hour light/dark cycle. Male LKO, littermate controls (*Kiss1r*$^{fl/fl}$) mice, and C57BL/6J mice (6 weeks old) were fed a high fat diet (HFD: 60% calories from fat, 0.28% from cholesterol, 20 % calories from carbohydrate, Research Diets catalog #D12492, New Brunswick, NJ) or regular control diet (RD: 5058 Purina PicoLab Mouse 20). Mice were group-housed and provided food and water ad libitum.

Administration of KP-analog

KP-analog, TAK-448 (referred to as KPA) was purchased from MedChemExpress. LKO or C57BL/6J male mice (5-6 weeks of age) were maintained on a HFD or RD for 6 weeks and then fasting blood glucose was measured. Alzet mini-osmotic pump model 2004 containing KPA (0.3 nmol/hr) (32) or PBS (vehicle controls) were inserted into the subcutaneous flanks of mice, as previously described (31, 32). Duration of the drug administration was five weeks, during which various metabolic tests were performed as described below, after which animals were euthanized and tissue collected for analysis. For knockdown of hepatic AMPK, C57BL/6J mice (6 weeks of age) were placed on HFD for 4 weeks then injected with AAV8-U6-M-PRKAA2-shRNA or AAV8-U6-M-scrambled-shRNA using validated sequences (Vector Biolabs, 5x10^{11} genome copies/mouse). Mice were kept on HFD for another three weeks before administration of KPA or Vehicle for 6 weeks, and then euthanized; off-target effects were not observed.

Diet Induced Animal Model of Non-Alcoholic Liver Disease (DIAMOND) mice were purchased from Sanyal Biotechnologies. This is a isogenic mouse strain derived from C57BL/6J &129S1/Svlm) that upon starting HFD and sugar water recapitulates human NAFLD (47). Male DIAMOND mice (8 weeks of age) were placed on a ‘Western’ high fat diet (42% calories from fat, 0.2% from cholesterol, 42.7% calories from carbohydrate, 15.2% calories from protein; Diet # TD.88137) in addition to sugar water (23.1 g/L of fructose and 18.9 g/L of glucose) for 33 weeks to induce advanced disease with Stage 3 fibrosis (47). KPA or Vehicle was then administered for 6 weeks while mice were maintained on diet, prior to euthanization.

Metabolic Tests and Metabolic Cage Assessments

Blood glucose measurements using a glucometer (Bayer Contour) were performed from a small nick in the lateral tail vein. For glucose tolerance tests (GTT), mice were fasted for 12 hours and then injected intraperitoneal with D-glucose (1g/kg). For the insulin tolerance tests (ITT), mice were fasted for 6 hours and then injected intraperitoneally with insulin (0.5 U/kg; Novo Nordisk). Mice were individually housed in an 8-chamber Comprehensive Laboratory Animal Monitoring System (CLAMS) with controlled light and feeding. Carbon dioxide output, oxygen uptake, respiratory ambulatory movement, and feeding were recorded over a 4-day period.

Quantitative Real-Time PCR (qPCR)

Total RNA was extracted from mouse tissues or hepatocytes using TRIzol reagent (ThermoFisher). RNA was extracted using RNeasy (Qiagen) from human liver biopsies purchased from Research Biobank Human Liver Sekusui XenoTech (**Supplemental Table 1**). Reverse transcription was done according to manufacturer's instructions using iScript RT Supermix (Bio-Rad). Gene expression was determined using SYBR green real-time qPCR (RT-qPCR) as described previously (85-87,70) using primers (**Supplemental Table 2**).

Immunoblot Analysis.

Immunoblot assays were conducted as described previously (62, 86). Mouse liver tissue or primary hepatocytes were homogenized in RIPA lysis buffer containing proteases and phosphatases inhibitors, centrifuged at 4° C and protein expression in supernatant was analyzed by Western blot analysis. Lysates from human NAFLD/NASH liver biopsies were purchased from Research Biobank Human Liver Sekusui XenoTech. Proteins were separated using SDS-PAGE and probed

using the antibodies (see Supplemental Table 3). Mouse anti-GAPDH, anti-vinculin 1:1000, anti-Tubulin, or mouse anti-Lamin were used for loading controls. Protein was then incubated for 1 hour in horseradish peroxidase (HRP)-conjugated rabbit 1:2500 (Cell Signaling Technology 7074S) or mouse 1:2500 (Cell Signaling Technology 7076S) secondary antibody. Blots were imaged by chemiluminescence with ChemiDocTouch imaging system (Bio-Rad) using SuperSignal and West Dura Extended Duration Substrate (Thermo Scientific). Protein levels were quantified using Image Lab Software (Bio-Rad).

Kisspeptin, Triglycerides, ALT, FFA, Glycerol, Insulin, Cholesterol, IL-1α, IL-1β, Ketone β-Hydroxybutyrate and Hydroxyproline Measurements: All assays were done according to manufacturer's instructions. Plasma kisspeptin levels were measured using KISS1 ELISA Kit (Antibodies online, Catalog # ABIN425747). Serum and liver triglycerides were measured using the triglyceride quantification kit (MBL international Catalog # JM-K622-500). ALT levels were measured using Liquid ALT (SGPT) Reagent (Catalog # A7526). Cholesterol levels were measured using the Cholesterol Liquid Reagent (Pointe Scientific, Catalog #C7510). Serum glycerol levels were measured using the Glycerol Assay (Sigma, Catalog #MAK117-1KT). Free fatty acids were measured using Free Fatty Acid Quantification Kit (Sigma, Catalog #MAK044). Insulin was measured using Insulin Mouse Ultra Sensitive ELISA (Crystal Chem, Catalog # 90080). IL-1α was measured using Mouse IL-1 alpha/IL-1F1 Quantikine ELISA Kit (R&D Systems, Catalog # MLA00). IL-1β was measured using Mouse IL-1 beta/IL-1F2 Quantikine ELISA Kit (R&D Systems, Catalog # MLB00C). Ketone β-Hydroxybutyrate was measured using β-Hydroxybutyrate (Ketone Body) Colorimetric Assay Kit (Cayman, Catalog #

700190). Hydroxyproline was measured using Hydroxyproline Assay Kit (Sigma, Catalog # MAK008).

Isolation of primary mouse hepatocytes

Mouse primary hepatocytes were isolated as described (56). Briefly, 8-10 week old mice were anesthetized, livers cannulated via the hepatic portal vein and perfused with Kreb's Ringer containing EGTA followed by a second Kreb's Ringer containing $CaCl_2$ and Liberase ™ Roche to dissociate the tissue. Cells were filtered and resuspended in William's Media E (Sigma) with 10 % FBS (Sigma), 200 nM dexamethasone (Sigma), Penicillin-Streptomycin (10,000 U/mL) and 2 mM L-glutamine (Fisher). Hepatocytes were plated at a density of 3×10^5 cells on 6-well collagen (Sigma) coated plates and left to recover overnight. Cells were serum starved for 3 h prior to experiments. Compound C was purchased from Sigma Millipore (catalog # 171261) and experiments were done as described (102). YM-254890 was purchased from Fisher (catalog #501490015) and experiments were done as previously described (67).

HepaRG cells

Human HepaRG cells were purchased from Thermo Fisher Scientific and cultured according to vendor's instructions. Briefly, cells were grown to confluence in Williams E Media supplemented with 10% fetal bovine serum, Penicillin-Streptomycin (10,000 U/mL), 2 mM L-glutamine, 5 microgram/mL Insulin (Humulin R.), 50 µM hydrocortisone hemisuccinate.

Triglyceride Synthesis

Free fatty acids (150 µM oleic and 150 µM palmitic acid) was conjugated with 2% BSA. Cells were loaded with FFA post isolation and left to recover overnight prior to serum starvation, and then treated with KP-10 (R&D Systems) or KPA (MedChemExpress). TGs were measured using the triglyceride quantification kit as per manufacturer's instructions (MBL international Catalog # JM-K622-500).

Immunofluorescence

Immunofluorescence studies were done, as described (85). Cells were fixed in formalin, permeabilized with 0.2% Triton X, and stained as described. Cells were incubated with KISS1 Antibody (EMD Millipore, 1:250) followed by goat anti Rabbit-AF555 (Invitrogen; 1:400). Nuclei were stained with Hoechst (Invitrogen; 1:10000). Images were acquired using a Zeiss LSM 700 laser scanning microscope.

Immunohistochemistry

Mouse livers were processed for histology by the Research Pathology Services at Rutgers University. Immunohistochemical analysis of mouse and human livers was reviewed by the pathologist, Dr. He Wang. Oil Red O staining was performed on cryostat sections of the frozen liver tissues to detect neutral lipids by the Wang laboratory and quantified using Halo image analysis (algorithm area quantification V2.1.3), as described (85, 103). Immunohistochemistry on human adult liver sections was performed as described (103). Paraffin sections of healthy human liver were purchased from OriGene Technologies, Inc. (MD, USA). Paraffin sections from NASH/NAFLD patients were obtained from archived liver tissue deposited at Robert Wood

Johnson University Hospital Pathology lab after diagnosis was confirmed by the pathologist. Following deparaffinization and heat induced antigen retrieval, slides were incubated with the rabbit polyclonal anti-GPR54 (Abcam, ab137483; 1:1000), followed by ImmPRESS™ HRP Anti-Rabbit IgG (Peroxidase) Polymer Detection Kit (Vector Laboratories).

Oxygen Consumption Rate (OCR)

OCR was measured using an Agilent Seahorse Biosciences extracellular flux analyzer (XFe24) as per the manufacturer's protocol using isolated primary mouse hepatocytes and human hepatic HepaRG cells. Briefly, cells were seeded at 2×10^4 cells per well in XF24 plates in William's E Media and were preincubated with 100 µM palmitate conjugated to BSA or BSA +/- KPA (3 nM) overnight at 37 °C and 5% CO_2. Cells were serum starved for 1 hr prior to the assay. Basal OCR measurements were made in Seahorse XF media with 0.5 mM glucose and 0.5 mM L-carnitine, treated with KPA (3 nM) in the presence of 100 µM palmitate or PBS. The results were analyzed using Wave software (Agilent Technologies).

Hormone Measurements

Serum testosterone and glucagon levels were measured at the University of Virginia Center for Research in Reproduction Ligand Assay and Analysis Core (Charlottesville, Virginia).

Statistics

Statistical significance between two groups was determined by unpaired two-tailed Student's t test. Unless indicated, for comparison among multiple groups, one-way analysis of variance (ANOVA)

followed by Dunnett's multiple comparisons test was used. A p-value <0.05 is considered to be statistically significant. Graphs were generated with GraphPad Prism version 8.3.1 (San Diego, CA).

Patient blood collection and plasma kisspeptin measurement

The study has been approved by the Institutional Review Board at Rutgers University and all study participants (males) provided written consent. Individuals with chronic medical conditions that may affect glucose or lipid metabolism including active malignancy, HIV infection, hepatitis B, hepatitis C, alcoholism, chronic pancreatitis, active viral/bacterial infection, severe cardiac or respiratory failure were excluded. Plasma KP immunoreactivity in healthy subjects (N=31) and patients with T2D (N=31) or with fatty liver (NAFL, N=34) or NASH (N= 25) was determined, as described (70, 100). A diagnosis of T2D was based on American Diabetes Association criteria (101). NAFL diagnoses were based on elevated AST (10-40 U/L) and ALT (9-46 U/L) levels in the absence of other causes of liver disease as well as the presence of hepatic steatosis on ultrasound. NASH diagnoses were based on histologic analysis revealing macrovesicular steatosis, lobular and portal inflammation and fibrosis. Briefly, blood (5 mL) was collected in BD Vacutainer K2 EDTA tubes (VWR International) from subjects recruited from Endocrinology (Dr. Ankit Shah's) and Hepatology (Dr. Vinod Rustgi's) clinics at Robert Wood Johnson Medical School in New Brunswick, NJ. Subjects were also recruited from Imperial College London/Imperial College Healthcare NHS Trust Metabolic and Hepatology clinics (Dr. Chioma Izzi-Engbeaya, Dr. Michael Yee, Dr. Pinelopi Manousou and Prof. Waljit Dhillo). This was reviewed and approved by the West London Research Ethics Committee (12/LO/0507) and was performed in accordance with

the Declaration of Helsinki. Blood was centrifuged for 10 min at 855 g and plasma was frozen immediately for storage. Plasma KP immunoreactivity was measured using an in-house radioimmunoassay in the Dhillo laboratory as described (100).

Supplemental Table 1: Clinical profile of commercially available male liver biopsies obtained from Research Biobank Human Liver Sekusui XenoTech

Diagnosis	Healthy (n=5)	NAFL/NASH (n=7)
Age (years)	51.6 ± 9	46.1 ± 5.5
BMI	25.6 ± 4.3	42.9 ± 11.4*
Macro fat % (Steatosis)	0	52.7 ± 26.6
Diabetes	0/5	2/7
Steatohepatitis	0/5	7/7
Fibrosis	0/5	2/7
Alcohol Consumption	4/5 None<br>1/5 Occasional	3/7 None<br>4/7 Occasional
Hepatitis B or C	Negative	Negative

*: $p<0.05$ significance compared to healthy

Supplemental Table 2: Primers

**Name**	**Forward**	**Reverse**
Acaca	ATGGGCGGAATGGTCTCTTTC	TGGGGACCTTGTCTTCATCAT
Acta2	ACTGGGACGACATGGAAAAG	GTTCAGTGGTGCCTCTGTCA
Aox	TGTCATTCCTACCAACTGTC	CCATCTTCTCAACTAACACTC
Cd36	GATGACGTGGCAAAGAACAG	TCCTCGGGGTCCTGAGTTAT
Col1a1	GAGAGAGCATGACCGATGGATT	TGTAGGCTACGCTGTTCTTGCA
Col1a2	GCAGGGTTCCAACGATGTTG	GCAGCCATCGACTAGGACAGA
Col3a1	TCCCCTGGAATCTGTGAATC	TGAGTCGAATTGGGGAGAAT
Col4a1	TTCGCCTCCAGGAACGACTA	AAACCGCACACCTGCTAATG
Cpt1a	AAACCCACCAGGCTACAGTG	TCCTTGTAATGTGCGAGCTG
Cpt2	AAGCCTCTCTTGAATGACAGC	CCAATGCCGTTCTCAAAATC
Cyp4a10	TTCCCTGATGGACGCTCTTTA	GCAAACCTGGAAGGGTCAAAC
Cyp4a14	CAAGACCCTCCAGCATTTCC	GAGCTCCTTGTCCTTCAGATGGT
Fasn	GGAGGTGGTGATAGCCGGTAT	TGGGTAATCCATAGAGCCCAG
Gk	ATCCGCTGGCTAAGAGACAACC	TGCACTGGGCTCCCAATAAGG
Il1a	CGCTTGAGTCGGCAAAGAAA	TGATACTGTCACCCGGCTCT
Il1b	CAACCAACAAGTGATATTCTCCATG	GATCCACACTCTCCAGCTGCA
Ip10	AAGTGCTGCCGTCATTTTCT	GTGGCAATGATCTCAACACG
Kiss1	AGCTGCTGCTTCTCCTCTGT	GCATACCGCGATTCCTTTT
Kiss1r	CTGCCACAGACGTCACTTTC	ACATACCAGCGGTCCACACT
Lfabp	GCAGAGCCAGGAGAACTTTGAG	TTTGATTTTCTTCCCTTCATGCA
Mcp1	AATGAGTAGCAGCAGGTGAGTG	GAAGCCAGCTCTCTCTTCCTC
Mip2	CCCAGACAGAAGTCATAGCCAC	GCCTTGCCTTTGTTCAGTATC
Mmp2	TCTGCGATGAGCTTAGGGAAAC	GACATACATCTTTGCAGGAGACAAG
Mmp13	AGAAGTGTGACCCAGCCCTA	GCGCAAGAAGAATCTGTCTTT
Opn	GACAACAACGGAAAGGGCAG	GATCGGCACTCTCCTGGCT
Pgc1a	TATGGAGTGACATAGAGTGTGCT	CCACTTCAATCCACCCAGAAAG
Pparg	CGACATGAGTTCCTTTATGATGGG	TGTGATCTCTTGCACGGCTT
Prkaa2	TCCAGCACAGCTGAGAACCA	GGGATGCCGAGGACAAAGT
Timp1	CCTTGCAAACTGGAGAGTGACA	AGGCAAAGTGATCGCTCTGGT
Tnfa	CGTCAGCCGATTTGCTATCT	CGGACTCCGCAAAGTCTAAG
Tgfb	TGACGTCACTGGAGTTGTACGG	GGTTCATGTCATGGATGGTGC
Rpl13a	GCTGCTCTCAAGGTTGTTCG	CCTTTTCCTTCCGTTTCTCC

Srebp1c, *Aqp3, Aqp9, Gpat2, Agpat2, Dgat1, Dgat2, Mogat1, Gpam, Ucp1, Ucp2* and mouse and human TBP primers were purchased from BioRad.

Supplemental Table 3: Antibodies

Antibody Name	Source	Company	Catalog #
ACC	Rabbit	CST	4190
AMPK	Rabbit	CST	2532
AMPKa1	Rabbit	CST	5832
AMPKa2	Rabbit	Abcam	3760
ATGL	Rabbit	CST	2138
COL1A1	Rabbit	CST	91144
COX1	Rabbit	CST	62101
DGAT1	Rabbit	Novus Biologicals	41487
FAS	Rabbit	CST	3180
GAPDH	Mouse	Genetex	627408
GK	Rabbit	Abcam	126599
GLYCEROL KINASE	Rabbit	Abcam	126599
GPAT1	Rabbit	Novus Biologicals	76907
HSL	Rabbit	CST	4107
IL1B	mouse	CST	12242S
KISS1	Rabbit	Proteintech	18375-1-AP
KISS1R	Rabbit	Abcam	137483
MMP13	rabbit	Thermo fisher	TA506688
MMP2	Rabbit	Abcam	228402
MMP9	Rabbit	Abcam	228402
MOGAT1	Rabbit	Abcam	81177
NFKB	Rabbit	CST	8242
pACC (ser79)	Rabbit	CST	11818
pAMPK (Thr172)	Rabbit	CST	2535
pATGL	Rabbit	Abcam	135093
pHSL	Rabbit	CST	45804
pNFKB	Rabbit	CST	3033
PPARG	Rabbit	CST	2443
PPARG	Mouse	Abcam	133625
pPPARG (Ser112)	Rabbit	Invitrogen	PA5-36763
SMA	Rabbit	CST	19245S
TGFB	Mouse	R&D Systems	MAB1835
TUBULIN	Mouse	CST	86298S
VDAC	Rabbit	CST	4661
VINCULIN	Mouse	Bio Rad	MCA465GA

GUZMAN ET AL SUPPLEMENTAL FIGURE 1

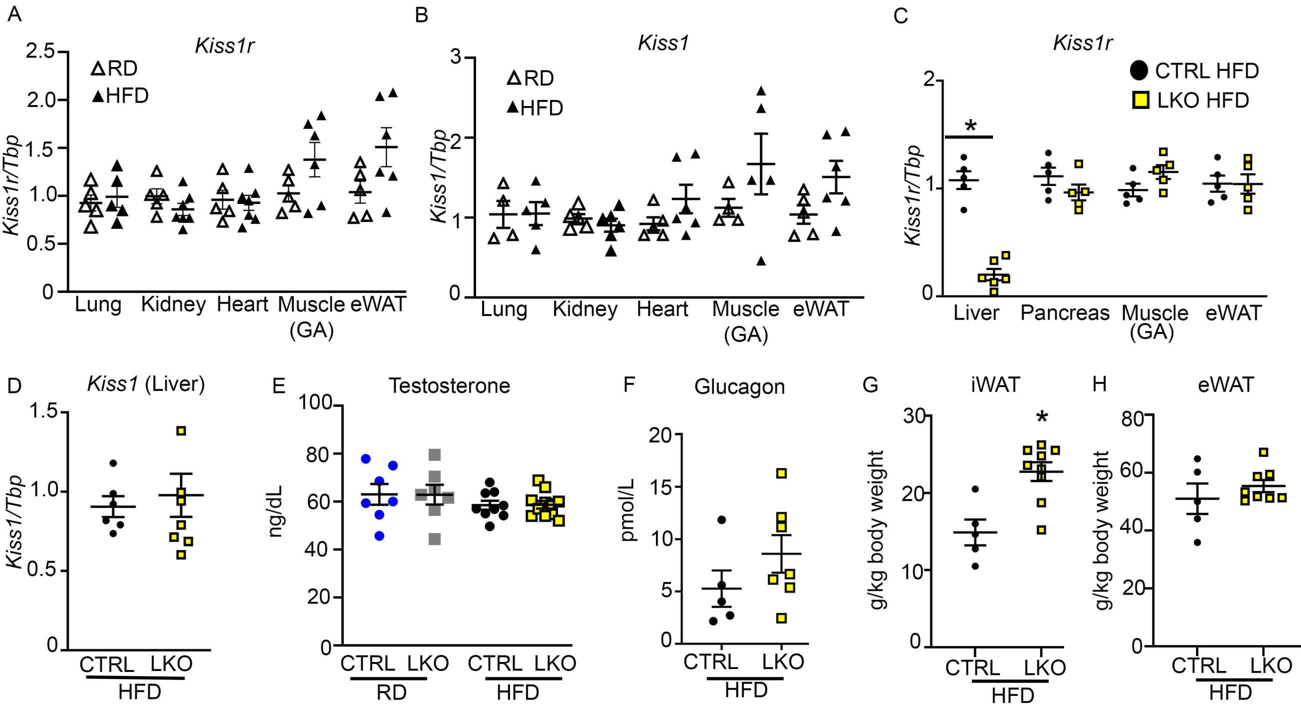

Supplemental Figure 1. (A-D) Relative mRNA expression of indicated genes by RT-qPCR; GA muscle: gastrocnemius muscle. (E) Serum testosterne levels. (F) Plasma glucagon levels. Weight of (G) inguinal white adipose tissue (iWAT) and (H) epididymal white adipose tissue (eWAT). Mean +/- SEM shown. Student's unpaired t-test or one-way ANOVA fullowed by Dunnett's post-hoc test; *p<0.05 versus respective controls.

GUZMAN ET AL SUPPLEMENTAL FIGURE 2

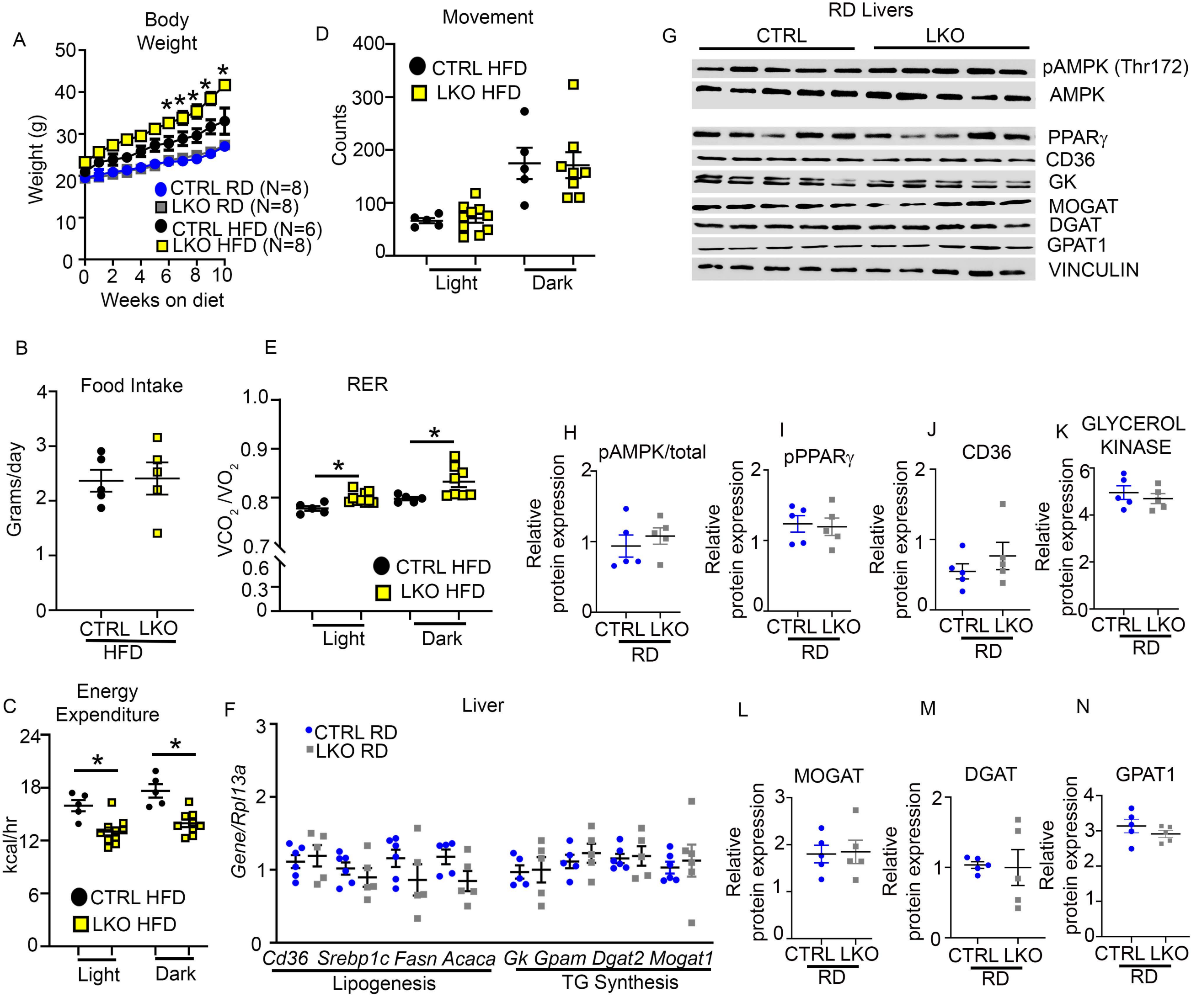

Supplemental Figure 2. (A) Body weight of male LKO and littermate controls (CTRL) during the first 10 weeks on diet. (B) Daily food intake (averaged over 4 days), assessed by CLAMS. CLAMS analysis displaying (C) energy expenditure, (D) ambulatory activity, (E) respiratory exchange ratio (RER). (F) Expression of indicated genes by RT-qPCR analysis in LKO and CTRL mice maintained on regular diet (RD) for 20 weeks. (G) Representative Western blots and (H-N) densitometric analyses of blots in (G). Mean +/- SEM shown. Student's unpaired t-test or one-way ANOVA followed by Dunnett's post-hoc test; *p<0.05 versus respective controls.

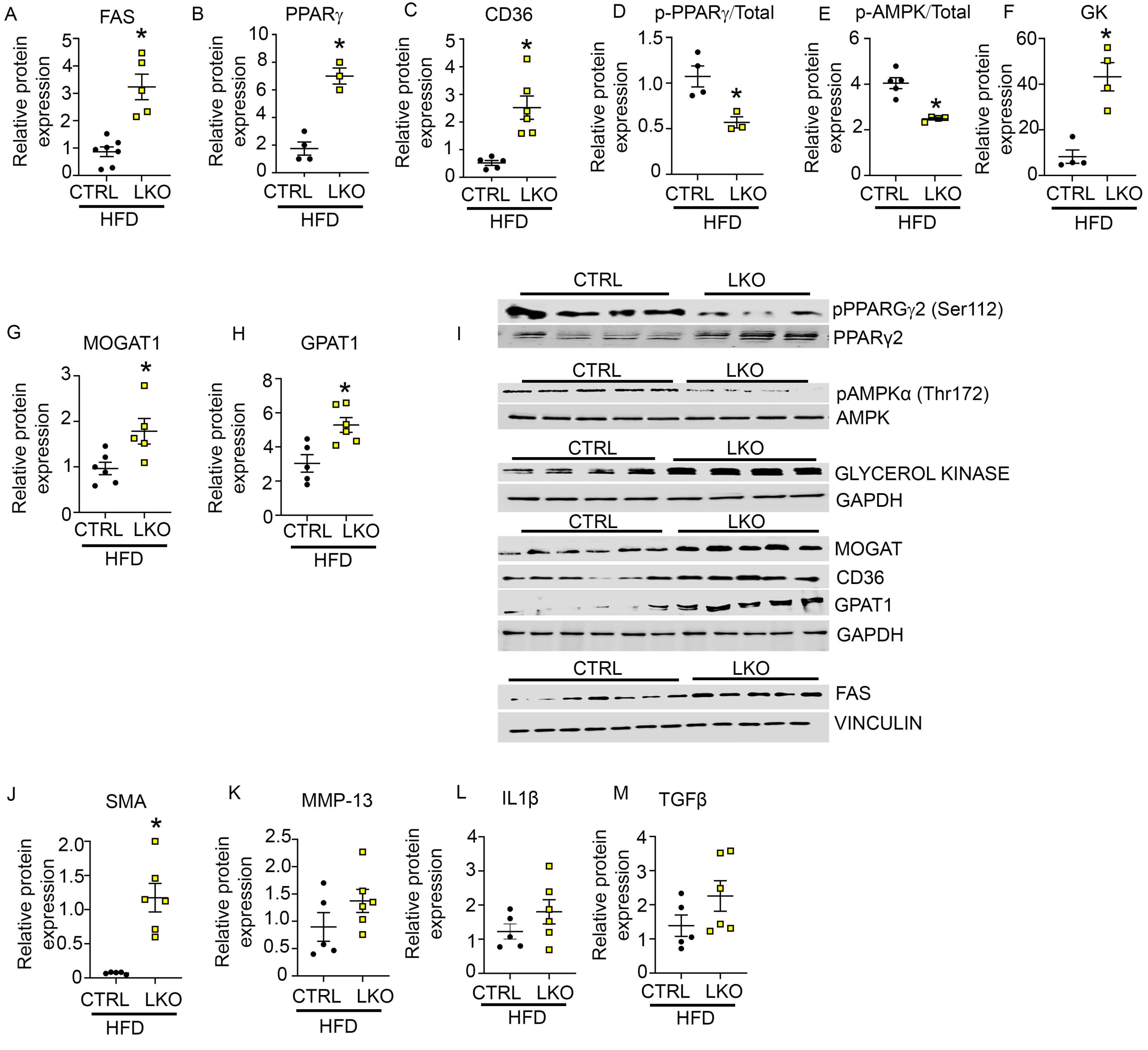

Supplemental Figure 3. Densitometric analyses of blots in Figure 2B (A-H). Full blots from Figure 2B shown in (I). Densitrometric analyses of blots in Figure 3J (J-M). Mean +/- SEM shown. Student's unpaired t-test *p<0.05 versus respective controls.

GUZMAN ET AL SUPPLEMENTARY FIGURE 4

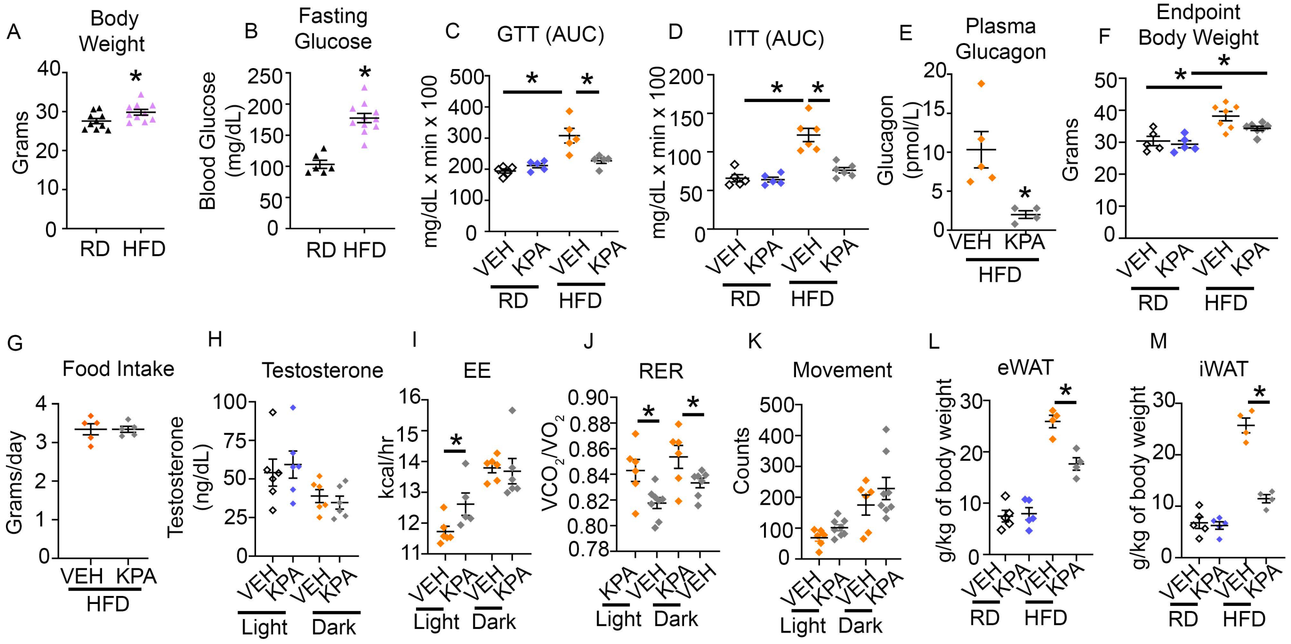

Supplemental Figure 4. C57BL/6J male mice were placed on RD or HFD for 6 weeks. (A) Body weight and (B) fasting glucose levels prior to treatment with Vehicle (VEH, PBS) or KPA. (C, D) Area under the curve (AUC) of blood glucose levels shown in GTT and ITT, respectively shown in Figure 4B, C. (E) Plasma glucagon levels, 5 weeks post treatment. (F) Endpoint body weight (11 weeks on diet). (G) Daily food intake measured by CLAMS. (H) Serum testosterone levels, 5 weeks post treatment. CLAMS analyses showing (I) energy expenditure (EE) (J) respiratory exchange ratio (RER) and (K) ambulatory activity of mice. Weight of white adipose tissue (L) epididymal (eWAT) and (M) inguinal (iWAT). Mean +/- SEM shown. Student’s unpaired t-test *p<0.05 versus respective controls.

GUZMAN ET AL SUPPLEMENTARY FIGURE 5

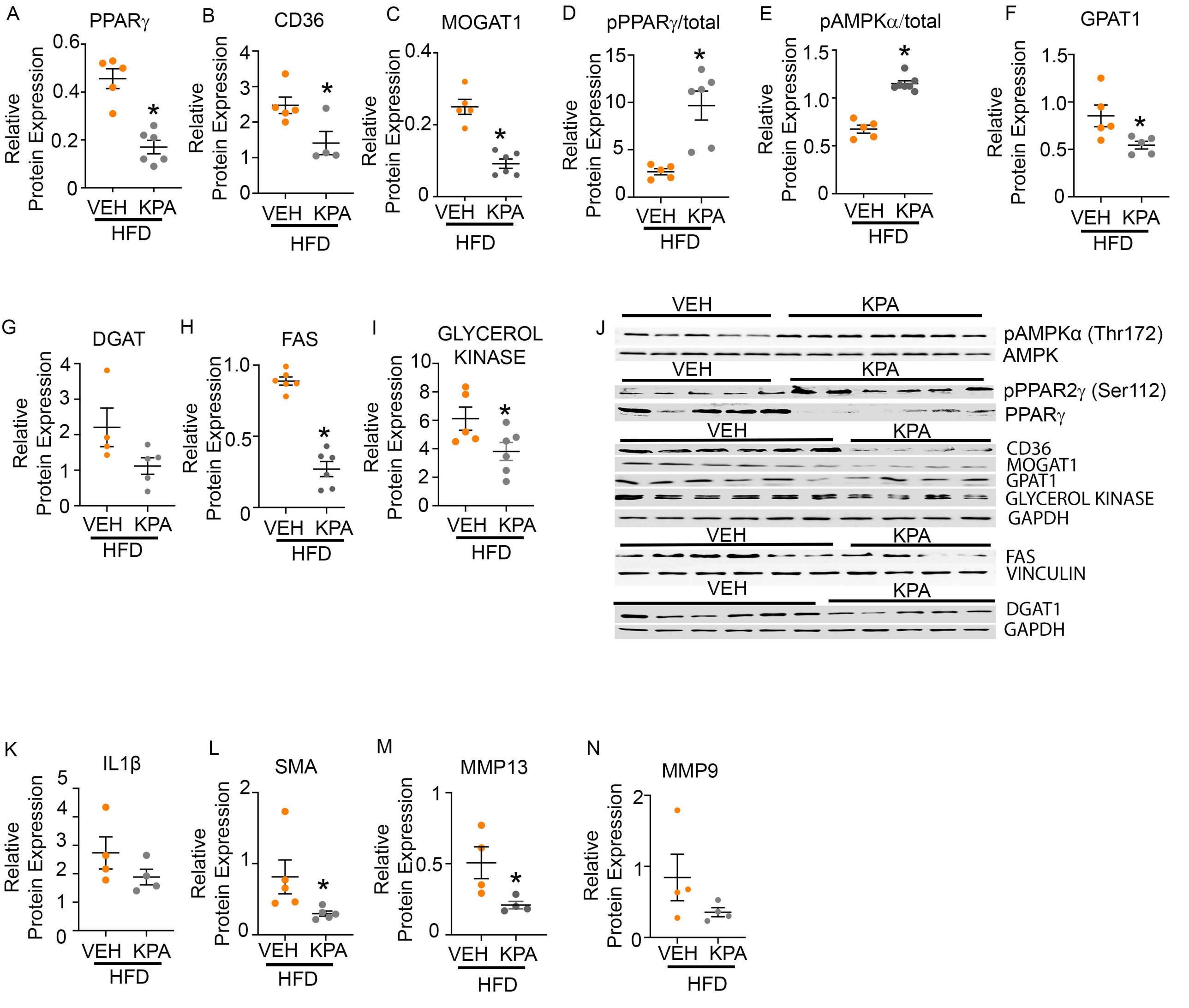

Supplemental Figure 5. (A-I) Densitometric analyses of blots in Figure 5A and (J) showing full blots (Figure 5A). (K-N) Densitometric analyses of blots in Figure 5G. Mean +/- SEM shown. Student's unpaired t-test *p<0.05 versus respective controls.

GUZMAN ET AL SUPPLEMENTARY FIGURE 6

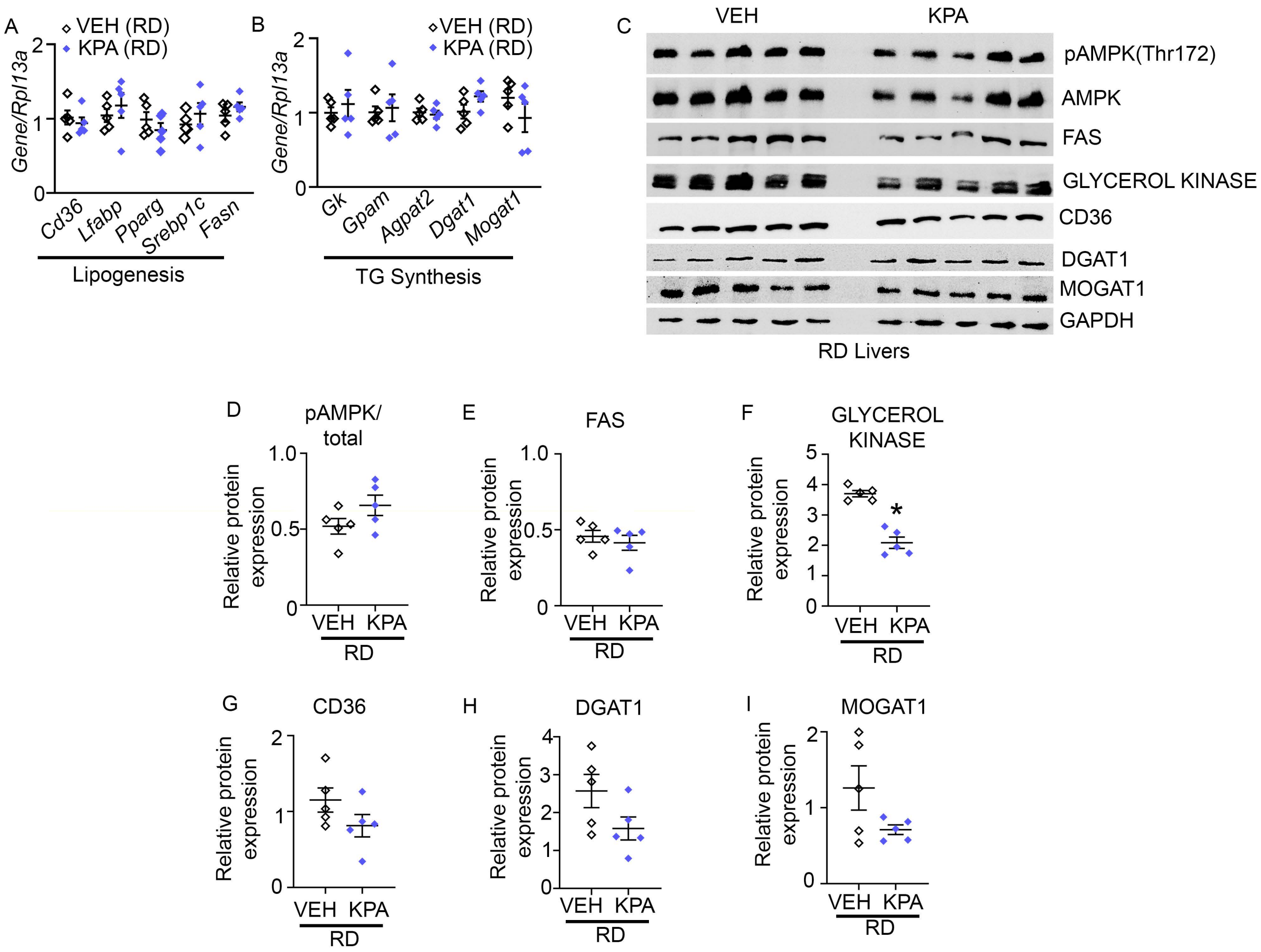

Supplemental Figure 6. C57BL/6J male mice were placed on RD for 6 weeks then VEH or KPA was administered for 5 weeks while maintained on diet. (A, B). Relative mRNA expression of indicated hepatic genes by RT-qPCR. (C) Representative Western blot analysis of indicated proteins.and densitometric analyses of blots shown in (D-I). Mean +/- SEM shown. Student's unpaired t-test *p<0.05 versus respective contro*l*s.

GUZMAN ET AL SUPPLEMENTARY FIGURE 7

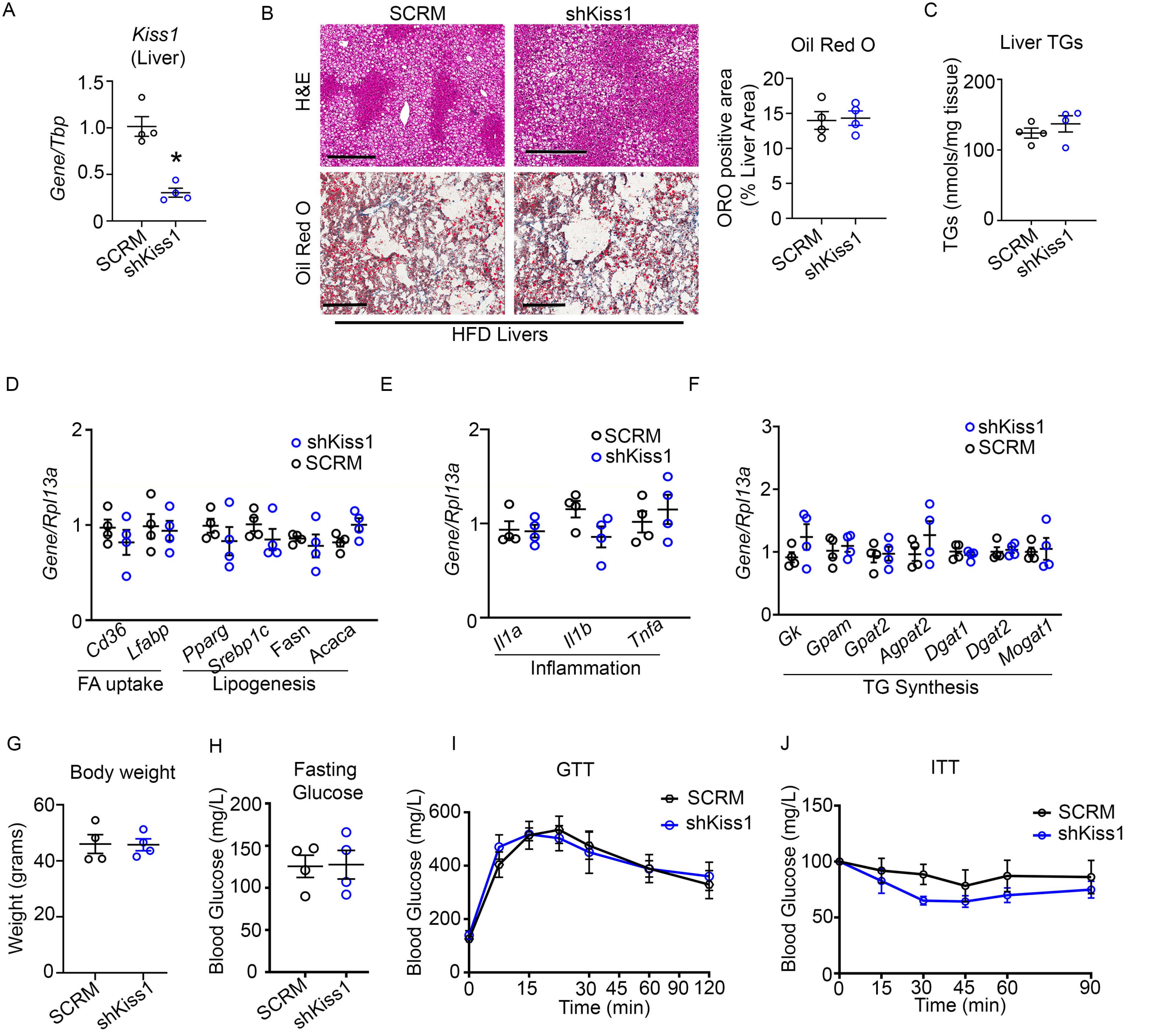

Supplemental Figure 7. C57BL/6J mice were placed on HFD for 10 weeks then injected with AAV8-U6-scrmb-shRNA (SCRM) or AAV8-U6-mKiss1-shRNA (shKiss1). (A) Hepatic *Kiss1* mRNA levels showing knockdown. (B) Representative histology of liver sections Oil Red O staining quantification shown in graph. Scale bar: 300 μm (C) Liver triglycerides (TGs). (D-F) Relative mRNA expression of indicated hepatic genes. (G) Endpoint bodyweight. (H) Fasting blood glucose levels. Blood glucose levels during (I) GTT and (J) ITT; (N=4 for SCRM or shKiss1 cohorts). Mean +/- SEM shown. Student's unpaired t-test or one-way ANOVA followed by Dunnett's post-hoc test. *p<0.05 versus respective controls.

GUZMAN ET AL SUPPLEMENTARY FIGURE 8

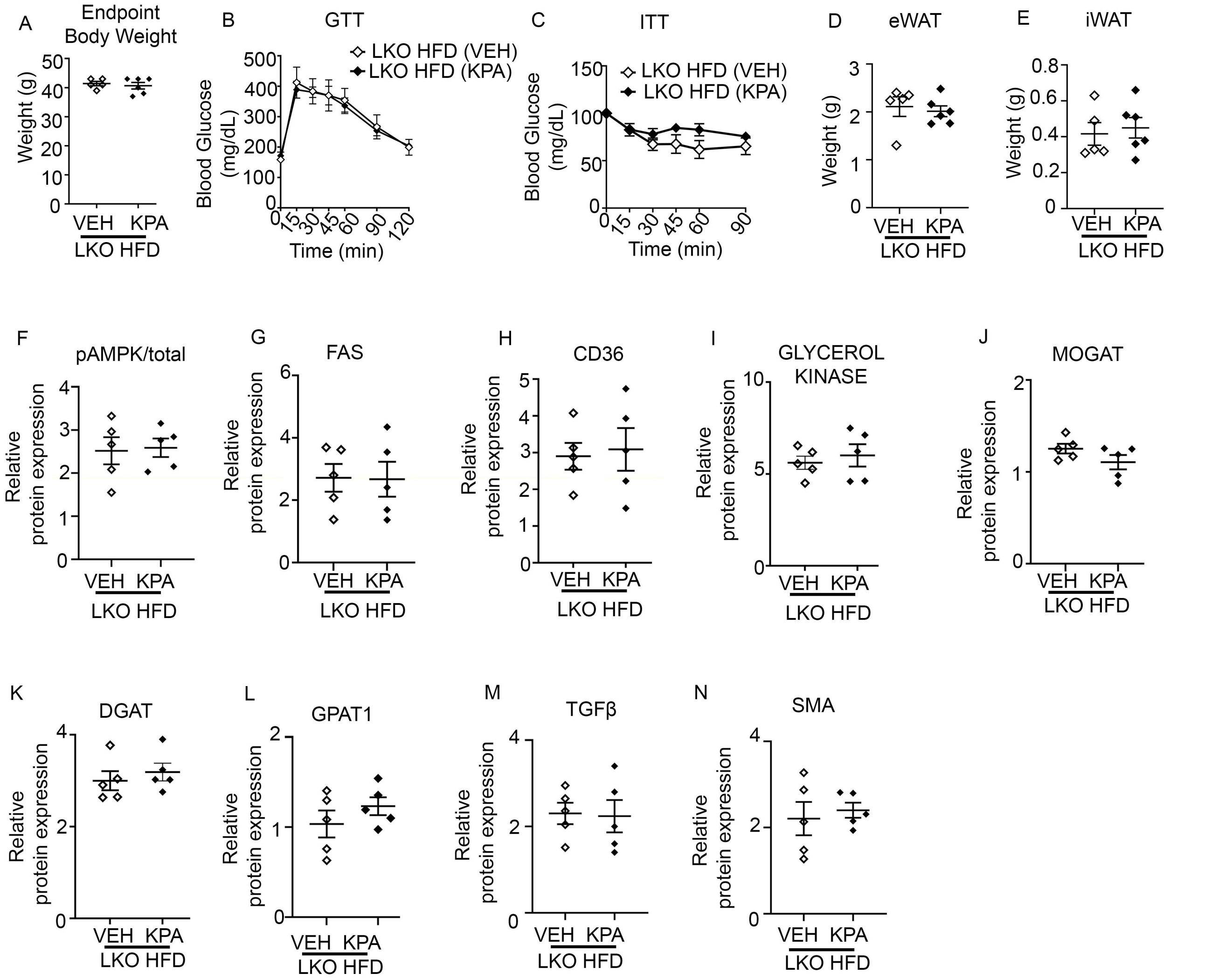

Supplemental Figure 8. Hepatic *Kiss1r* knock-out mice were placed on HFD for 6 weeks, then treated with Vehicle (VEH: PBS) or KPA. Mice were maintained on HFD for another 6 weeks. (A) Endpoint body weights of mice on HFD for 12 weeks (6 weeks after the start of treatment). Blood glucose levels during (B) GTT and (C) ITT. [N=5 LKO HFD (VEH); N=6 LKO HFD (KPA)].Weight of (D) eWAT and (E) iWAT. (F-N) Densitometric analyses of Western blots shown in Figure 6J. Mean +/- SEM shown. Student's unpaired t-test or one-way ANOVA followed by Dunnett's post-hoc test.*p<0.05 versus respective controls.

GUZMAN ET AL SUPPLEMENTAL FIGURE 9

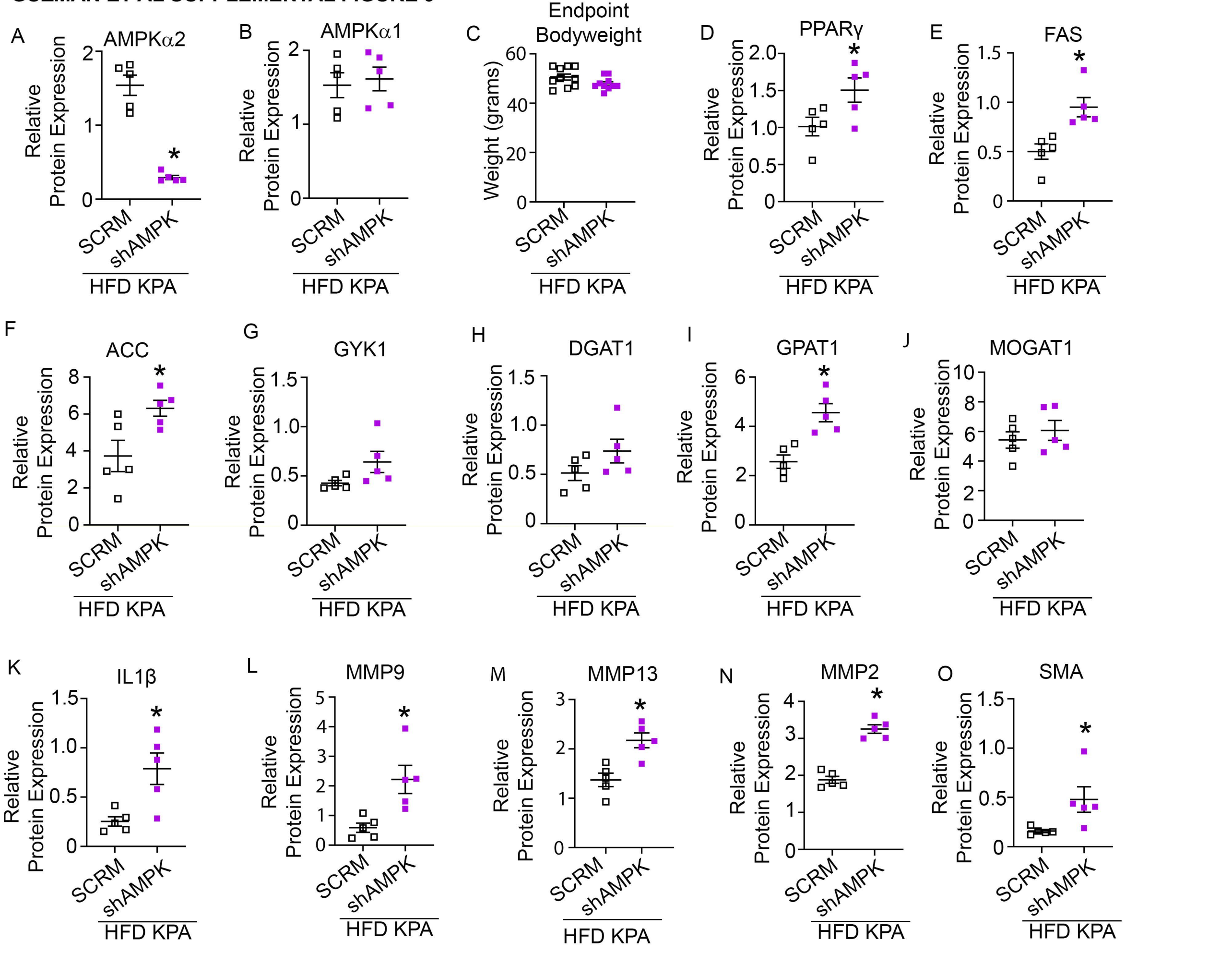

Supplemental Figure 9. Densitometric analyses of blots in Figure 7B (A, B), Figure 7I (D-J) and Figure 7M (K-O) showing the expression of the indicated proteins. (C) Endpoint bodyweight of SCRM and shAMPK mice on HFD for 13 weeks (6 weeks after start of treatment). Mean +/- shown.Student’s unpaired t-test *p<0.05 versus respective controls.

GUZMAN ET AL SUPPLEMENTAL FIGURE 10

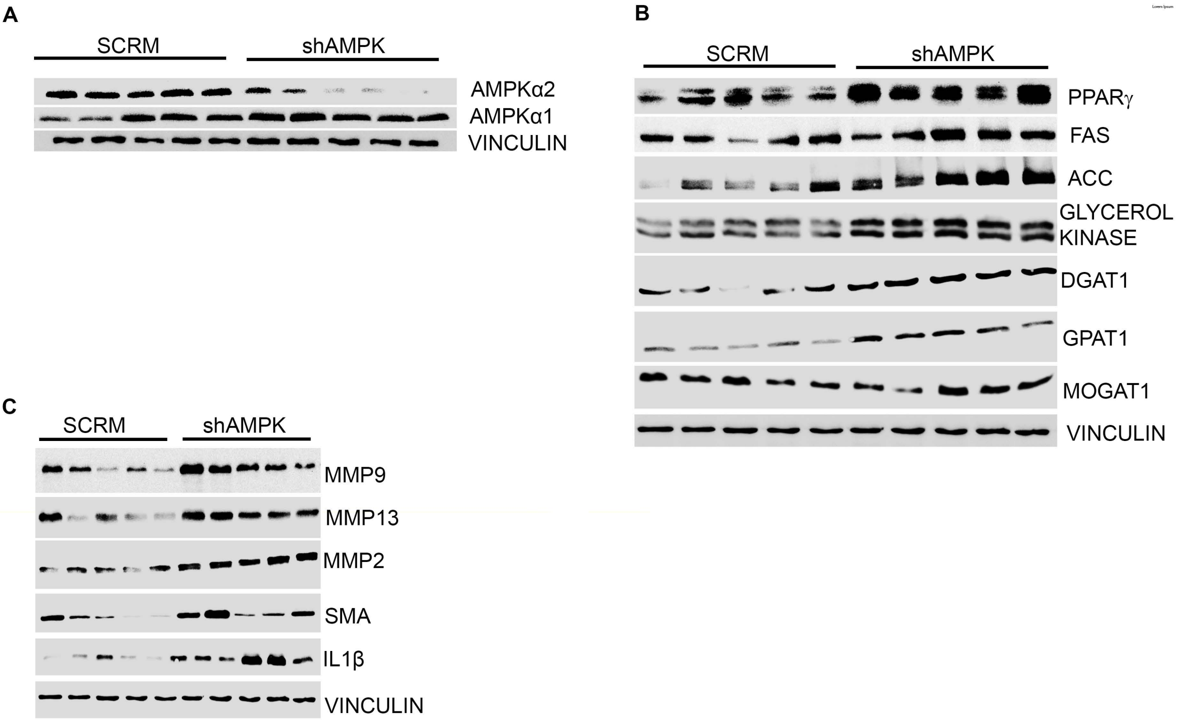

Supplemental Figure 10. Full blots from (A) Figure 7B, (B) Figure 7I. and (C) Figure 7M.

SUPPLEMENTAL FIGURE 11

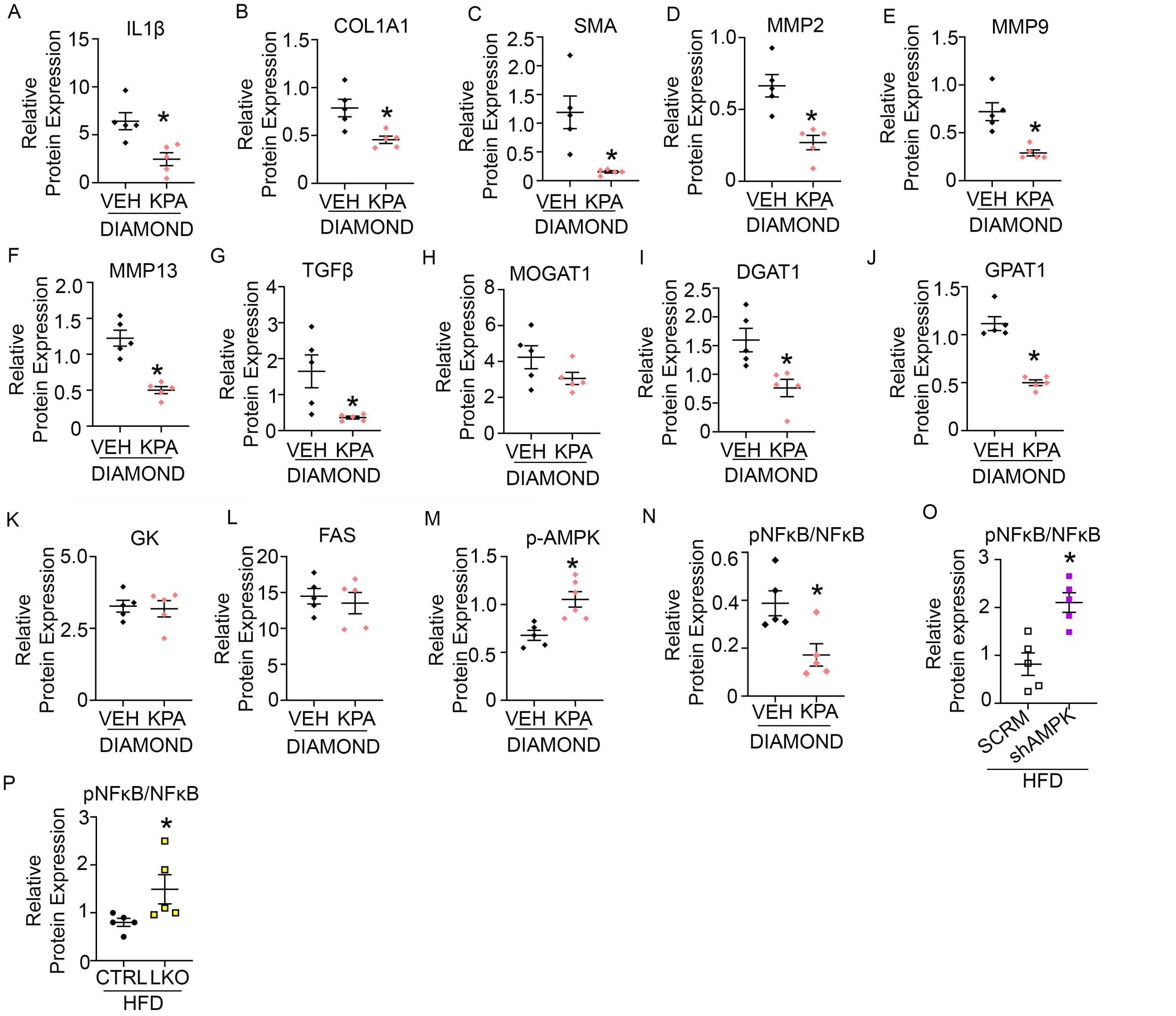

Supplemental Figure 11. Densitometric analysis of blots in Figure 8G (A-G) and Figure 9 G (H-L), Figure 9H (M), Figure 9K (N), Figure 9L (O), Figure 9M (P) showing the expression of the indicated proteins. Mean +/- SEM shown. Student's unpaired t-test *<0.05 versus respective controls.

SUPPLEMENTAL FIGURE 12

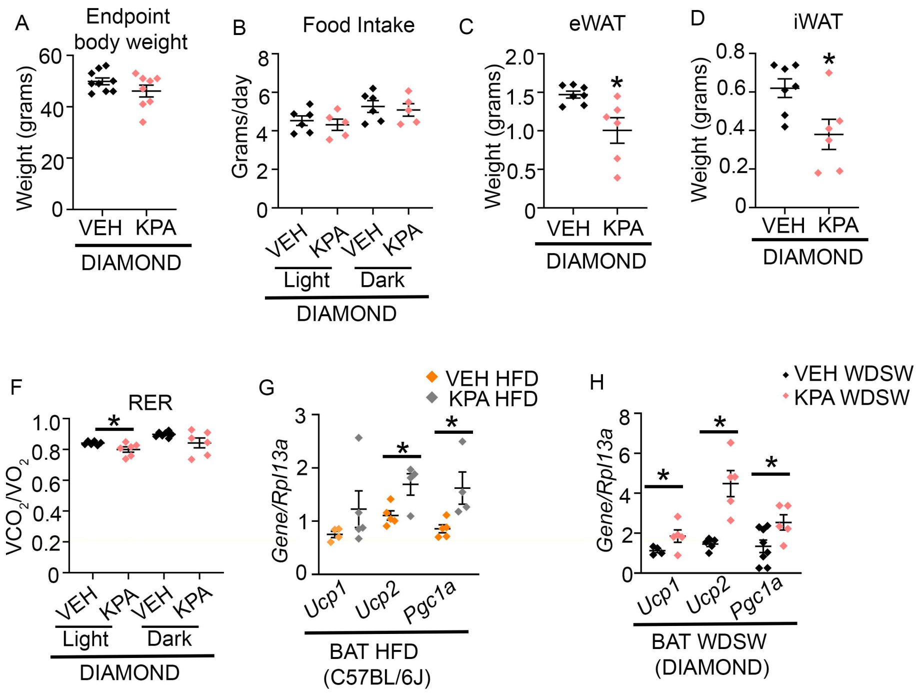

Supplemental Figure 12. (A) Endpoint bodyweight of DIAMOND mice on Western diet and sugar water (WDSW). (B) Daily food intake (averaged over 4 days), assessed by CLAMS. Weight of (C) epididymal white adipose tissue (eWAT) and (D) inguinal white adipose tissue (iWAT). CLAMS analysis displaying (E) energy expenditure and (F) respiratory exchange ratio (RER). (G, H) Expression of indicated genes by RT-qPCR in brown adipose tissue (BAT). Mean +/- SEM shown. Student's unpaired t-test *<0.05 versus respective controls.

GUZMAN ET AL. SUPPLEMENTAL FIGURE 13

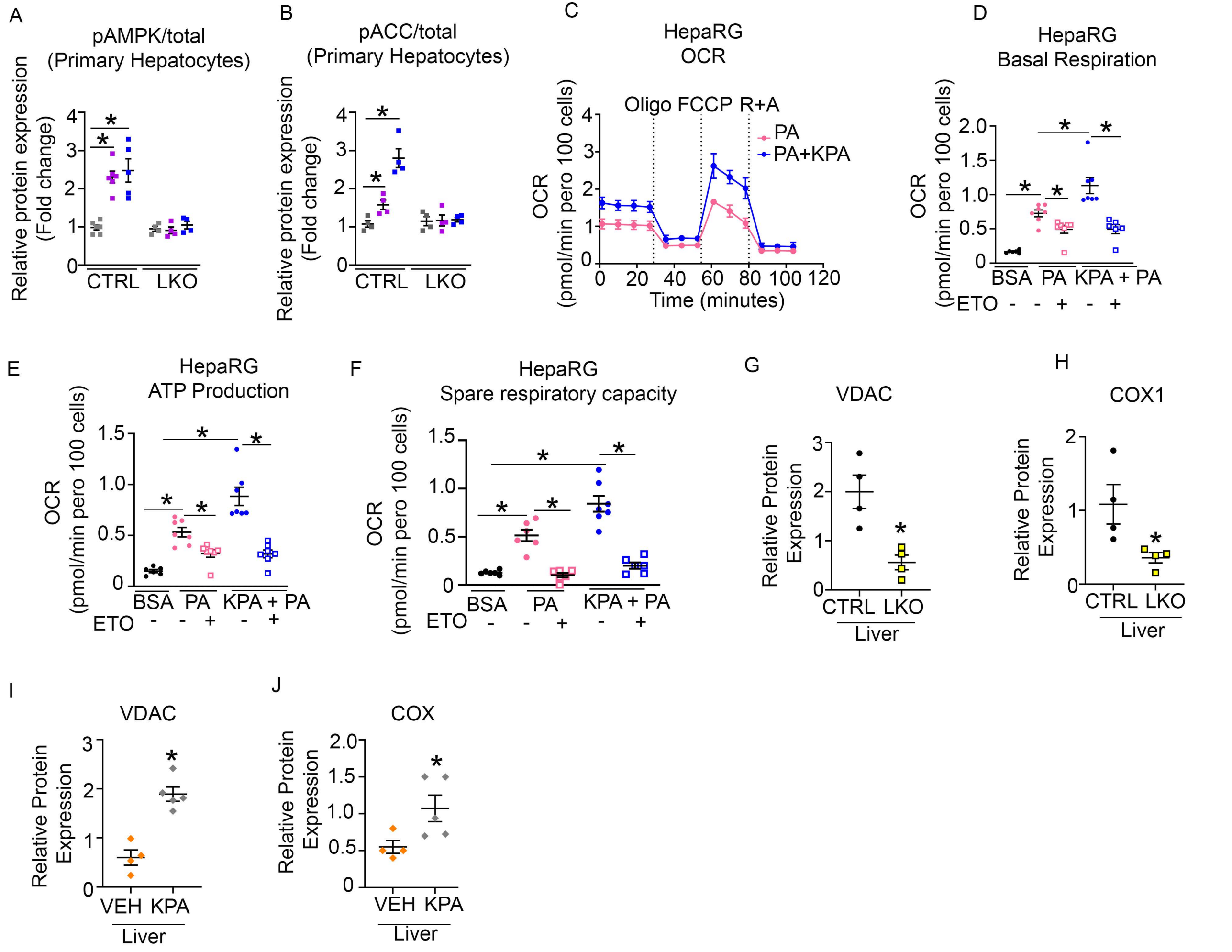

Supplemental Figure 13. (A-B) Densitometric analysis of blots from Figure 10E. (C) Seahorse traces of OCR in human hepatic HepaRG cells following sequential treatment with 1 μM oligomycin (Oligo), 1 μM FCCP and 0.5 μM of rotenone and antimycin A (R+A), respectively. Cells treated with 100 μM Palmitate or BSA in the presence or absence of KPA (3 nM). (D) Basal respiration, (E) ATP production and (F) spare respiratory capacity of cells. Densitometric analysis of Western blots in Figure 11E (G, H). and Western blots in Figure 11F (I, J). Mean ± SEM shown. Student's unpaired t-test, or one-way ANOVA followed by Dunnett's post-hoc test; *$p < 0.05$ compared to controls.
